# Looking for hotspots of marine metacommunity connectivity: a methodological framework

**DOI:** 10.1038/srep23705

**Published:** 2016-03-31

**Authors:** Paco Melià, Marcello Schiavina, Marisa Rossetto, Marino Gatto, Simonetta Fraschetti, Renato Casagrandi

**Affiliations:** 1 Dipartimento di Elettronica, Informazione e Bioingegneria, Politecnico di Milano, via Ponzio 34/5, 20133 Milano, Italy; 2 Consorzio Nazionale Interuniversitario per le Scienze del Mare, Piazzale Flaminio 9, 00196 Roma, Italy; 3 Dipartimento di Scienze e Tecnologie Biologiche ed Ambientali, Università del Salento, Strada Provinciale Monteroni, 73100 Lecce, Italy

## Abstract

Seascape connectivity critically affects the spatiotemporal dynamics of marine metacommunities. Understanding how connectivity patterns emerge from physically and biologically-mediated interactions is therefore crucial to conserve marine ecosystem functions and biodiversity. Here, we develop a set of biophysical models to explore connectivity in assemblages of species belonging to a typical Mediterranean community (*Posidonia oceanica* meadows) and characterized by different dispersing traits. We propose a novel methodological framework to synthesize species-specific results into a set of community connectivity metrics and show that spatiotemporal variation in magnitude and direction of the connections, as well as interspecific differences in dispersing traits, are key factors structuring community connectivity. We eventually demonstrate how these metrics can be used to characterize the functional role of each marine area in determining patterns of community connectivity at the basin level and to support marine conservation planning.

Seascape connectivity, broadly intended as the exchange of organisms across a heterogeneous mosaic of habitat patches^1^, is a key factor affecting the spatiotemporal dynamics of marine metapopulations and metacommunities^2^. The interaction between the physical environment and life-history, trophic and behavioural traits determines a range of connectivity patterns that shapes the assembly of biodiversity at different spatial scales^3^ and influences the response of metacommunities to environmental and anthropogenic disturbance^4^. Understanding how connectivity patterns emerge from physically and biologically-mediated interactions is therefore crucial to marine ecology and biodiversity conservation^5^,^6^,^7^. Because of the uneven spatial distribution of biodiversity in the sea, ensuring connectivity across protected areas is recognised as a major ecological principle to design effective networks of marine reserves^8^,^9^,^10^,^11^,^12^. A rule of thumb for planning is that reserves should exchange a sufficient number of propagules with each other^13^. However, devising operational rules to determine optimal spatial configurations is complicated by our limited knowledge of metacommunity connectivity.

Most studies on seascape connectivity have focused on one or two species, while studies on more species are relatively scarce^6^,^14^,^15^. Individual-based biophysical models provide a useful tool to complement the information gathered in the field^16^,^17^ and to contrast alternative hypotheses possibly emerging from the analysis of field data^18^,^19^. The cross-validation between modelling results and experimental data (including physical measures of flow speed and direction, assessment of α and β-diversity, estimates of genetic differentiation) can set the basis to understand the links between oceanography and ecology^20^,^21^,^22^,^23^. Nevertheless, biophysical models have been rarely^2^,^12^,^15^ applied to the analysis of metacommunity connectivity.

Increasing evidence shows that biological conservation should adequately consider the complexity of ecosystems across a range of spatial scales instead of focusing on single species^24^,^25^,^26^. Although conservation efforts were initially oriented toward single surrogate species with high cultural, economic or ecological importance, marine protected areas (MPAs) increasingly aim to protect biodiversity in a holistic fashion^27^. To establish MPA networks and assess their effectiveness, the concept of connectivity should be extended from a single-species to a community perspective^28^. The vast range of developmental patterns and behaviours exhibited by marine organisms, and their interaction with the physical environment, create complex connectivity patterns in space and time. Unravelling the linkages between these drivers is an important challenge, and building a conceptual framework for the interpretation of connectivity at the community level is essential to support the design of conservation strategies informed by a broader ecological perspective.

We explored community connectivity over a decade (2003–2013) within a region of about 250,000 km^2^ in the central Mediterranean, encompassing the Adriatic Sea and the northernmost part of the Ionian Sea (Fig. 1). We focused on a set of key species belonging to the same community: one primary producer (*Posidonia oceanica*), whose critical role as a habitat former in the Mediterranean is well documented^29^, and three fish species (*Sarpa salpa*, *Symphodus ocellatus* and *Scorpaena porcus*) occupying different trophic levels and characterized by different dispersing traits (Fig. 2). We built a set of individual-based biophysical models^16^ using a state-of-the-art physical engine and accounting for species-specific dispersal features. We ran Lagrangian simulations to investigate the dispersal ability of each species, its variation over the geographic domain under study, and how this affects connectivity across the sectors into which the suitable habitat was subdivided. For each species we derived dispersal kernels^30^ and connectivity matrices^21^ that were used to calculate a set of species-specific connectivity indicators. Connectivity was characterized by three metrics: *intensity* (the flux of propagules successfully moving from the suitable area of a source sector to the suitable area of a sink sector), *effectiveness* (the ratio between the flux of propagules successfully moving from/towards a sector and its capacity to donate/receive propagules), and *persistence* (the continuity of the flux throughout the years). We produced a connectivity matrix for each metric, thus providing substantial and complementary information to quantify connectivity between sectors. To characterize the functional role of each sector within the study area, we calculated the metrics cited above on the basis of fluxes originating from and received by the same sector (*retainer*), originating from a sector (*source*) and received by any other, and originating from any other and arriving to a sector (*sink*). Then, we aggregated species-specific indicators into a set of indices characterizing connectivity at the community level.

Finally, to exemplify how the proposed indices can inform marine conservation strategies, we used them to characterize the strength of community connectivity of each sector. To this end, we defined a connectivity score summarizing the capacity of each sector to simultaneously act as a retainer, source and sink of propagules for the considered community. Sectors with the highest connectivity score can be considered as *hotspots* of community connectivity, because they are able to play all the three functions at the same time and for all the considered species, deserving therefore high priority for protection.

## Results

### Species-specific connectivity

For each species, we derived two dispersal kernels: one measuring the net displacement of propagules from the origin to the destination, the other measuring the directed displacement (in sectors) along the coastline (Supplementary Fig. S1). Net displacement kernels (Fig. 3a) have in many cases a monotonically decreasing trend, although some show “holes” at intermediate distances. This can be due either to ocean circulation, preventing propagules from reaching destination sectors placed at specific distances, or to the absence of suitable conditions in those sectors. Dispersing potential varies widely, both among species and across the study area: the species with the lowest dispersal capacity in the Adriatic is *S. ocellatus*, whose maximum displacement ranges from less than 25–50 km (for propagules starting from Greece and Albania) up to 250 km (from Dalmatia and Apulia). In contrast, propagules of *S. salpa* are those dispersing farthest, covering distances as long as 500 km and up to 750 km. Net displacement is larger for propagules originating from Dalmatia, Apulia and, to a lesser extent, Albania. Notice that, for regions like Greece and the Gulf of Taranto, which are at the borders of the model domain, edge effects may affect estimates of maximum displacement; in fact, as propagules are tracked only as long as they remain within the model domain (i.e. north of 39°N), those going outside are classified as unsuccessful, and the resulting dispersal kernel may be (at least partially) truncated.

The geographic variation in the dispersal capacity of each species is made evident by the diversity of shapes displayed by dispersal kernels among regions. Figure 3b shows that propagule dispersal across the study area is strongly oriented: in almost all regions, directed displacements are more frequently distributed on the left side of the panel (i.e. towards negative values): such a structure reflects the counter-clockwise (cyclonic) flow of the oceanic circulation (Supplementary Fig. S2). Dispersal is prevalently oriented northwards along the eastern side of the Adriatic and southwards along the western side. Most propagules remain within less than 10 sectors from their origin; this is particularly true for *S. ocellatus*, for which the majority of propagules are retained within the sector of origin or in the nearest neighbours. Species with a wider dispersal capacity, such as *S. salpa* and *S. porcus*, are able to cross the Adriatic, mainly moving from east to west, with very few propagules moving in the opposite direction. Noticeably, the dispersal of *S. porcus* from south-eastern Adriatic regions (Albania and Montenegro) targets towards the coasts of the opposite side (Apulia and Gulf of Taranto) rather than those on the same side (Dalmatia).

Supplementary Figs S3–S14 show species-specific connectivity matrices and histograms indicating intensity, effectiveness and persistence of each sector as a retainer, source or sink of propagules. The matrices have a block structure, because not all sectors host suitable habitat. In particular, the cross-shaped grey area in the centre of the matrices indicates that propagules can neither start from (grey rows) nor arrive to (grey columns) any of the sectors located in the north-western Adriatic (from the Gargano peninsula up to the Po Delta and the Venetian Lagoon), where suitable conditions for *P. oceanica* are lacking. On the other hand, white matrix cells represent potential connections (because origin and destination sectors are both suitable) that, however, are not active, i.e. they were not followed by any Lagrangian particle. A number of coloured cells, indicating active connections, emerge from the white blocks: they are more abundant along the diagonal and the lower triangular part. As pointed out above, this reflects the fact that propagule dispersal is mainly driven by the major cyclonic meander flowing along Adriatic coasts. Connections following the cyclonic circulation are also characterized by higher persistence over time, while those in the opposite direction, as well as those connecting the two sides of the Adriatic, are generally more occasional.

Connectivity of *P. oceanica* is characterized by intense fluxes connecting the northern sectors of Adriatic Apulia with southern ones. Moderately intense connections link southern Apulia with the Gulf of Taranto, and locations along the whole Gulf with each other. Dalmatian coasts are also intensely cross-connected (from south to north). Bidirectional exchanges of propagules, though non-symmetrical in terms of intensity, also link the two sides of the basin. Retention is common and intense all over the suitable areas, as revealed by the warmer colour of the cells along the diagonal of the intensity matrices and by the histograms of retention intensity. Connectivity patterns of *S. salpa* remind those of *P. oceanica*, but extend over a wider geographical range, with propagules originating from Greece and Albania arriving up to northern Dalmatia, and fluxes connecting northern Apulia with the Gulf of Taranto up to its western side. Cross-Adriatic connections are more intense than those of *P. oceanica*, and link southern Apulia with Dalmatia, as well as Albania and Greece with the Gulf of Taranto and vice versa (although east-to-west connections are more numerous and intense than those in the opposite direction). On average, connections obtained for *S. salpa* are characterized by lower intensity and persistence compared with those for *P. oceanica*. In contrast, source and sink effectiveness of *S. salpa* are the highest among the four species. Connectivity patterns of *S. ocellatus* show strong retention and very confined dispersal across a narrow geographical range. Very occasional and weak connections link the two sides of the Adriatic, while moderately intense fluxes connect the southernmost sectors of Apulia with the eastern part of the Gulf of Taranto. Retention is, on average, more intense and persistent than that of *P. oceanica* and *S. salpa*, while source and sink intensity are intermediate between the two. Retention effectiveness is by far the highest among the four species, and source and sink effectiveness are the lowest. The most peculiar connectivity pattern is shown by *S. porcus*: most longitudinal connections, such as those between eastern and western Adriatic coasts, or between the two sides of the Gulf of Taranto, are mono-directional (as revealed by the asymmetry of the connectivity matrix), with particle fluxes directed only from east to west. Also, connections across the Dalmatian region are more fragmented compared with those of *P. oceanica* and *S. salpa*. Intensity and persistence are, on average, the lowest within the model community, and the same holds for retention and source effectiveness.

### Community connectivity

The aggregation of species-specific connectivity metrics into community connectivity metrics reveals how the interplay between oceanic circulation and different dispersing features shapes the dispersal of species assemblages. The set of species considered, with their specific life-history traits, has a critical influence on the resulting connectivity patterns. In the case of the model community under study, this emerges clearly by comparing connectivity patterns obtained considering three-species assemblages, in which only one of the two secondary consumers (either *S. ocellatus* or *S. porcus*) is included, along with the primary producer (*P. oceanica*) and the primary consumer (*S. salpa*), as well as the entire four-species assemblage. Figure 4 shows matrices of community connectivity intensity for the *Posidonia*-*Sarpa*-*Scorpaena* assemblage (panel a) and the *Posidonia*-*Sarpa*-*Symphodus* assemblage (panel b). Despite apparent similarities between the structures of the two matrices (such as the white block in the upper-right corner or the lower triangular structure of the block corresponding to Apulia) important differences are worth remarking. When *S. porcus* is included in the assemblage, the Gulf of Taranto is characterized by a dense system of connections from each side of the Gulf to the other (especially east to west) and a rich flux of propagules coming from southern Apulia. Instead, the spatial range of connectivity within that region is narrower when *S. porcus* is replaced by *S. ocellatus*. The short-ranged movement of *S. ocellatus* results in high retention and intense local connections within the Dalmatian region; in contrast, when only *S. porcus* is considered, connections across the region are less intense, more fragmented and limited to the northern part of the region. Yet, the most striking difference at the scale of the entire study area regards the role of Italian sectors as sinks of propagules coming from the other side of the Adriatic: when the assemblage includes *S. porcus*, there is a large number of active connections linking Dalmatia, Montenegro and Albania with the Gulf of Taranto and Adriatic Apulia, while when *S. ocellatus* is included the link between the two sides is limited to very few (and weak) connections from Albania to the Gulf of Taranto. Interestingly, in fact, for both assemblages the Tremiti Islands act as the only stepping stone for the dispersal of propagules from west to east.

Connectivity matrices for the entire (four-species) assemblage are shown in Supplementary Figs S15 (intensity), S16 (effectiveness), and S17 (persistence), along with the corresponding histograms of retention, source and sink strength. Community connectivity is apparently limited by the short-range dispersal of *S. ocellatus*, which confines community connections within relatively small neighbouring areas. Very few cross-Adriatic connections link Adriatic Apulia with southern Dalmatia, and Albania with the eastern Gulf of Taranto; they are characterized by very low intensity and persistence. From the perspective of the four-species assemblage, the Adriatic appears as subdivided into two major regions (the western and the eastern side of the basin), characterized by strong connectivity within each region and low connectivity between regions.

To make the information contained in the community connectivity matrices easier to read, and to provide synthetic indices that can support decision makers in site prioritization, we ranked the sectors according to the functional role they play in the region, thus assigning each of them a percentile rank with respect to its retention, source and sink strength. Function-specific percentile ranks were obtained by aggregating the corresponding intensity, effectiveness and persistence indices, as detailed in the Methods. The geographic distribution of percentile ranks for each functional role is displayed in Supplementary Figs S18 (retention strength), S19 (source strength) and S20 (sink strength). Percentile ranks were eventually aggregated into a *community connectivity score*, summarizing the capacity of each sector to act, at the same time, as a retainer, source or sink of propagules for the four species composing the assemblage. Figure 5a shows the cumulative distribution of percentile ranks (for each functional role) and of the community connectivity score. Differences between function-specific percentile ranks reveal that best retainers (i.e. sectors that are top-ranked in terms of retention intensity, effectiveness and persistence at the same time) are more numerous than best sources or best sinks for our four-species assemblage in the considered region. In particular, the most stringent functional role is that of acting as a sink. None of the sectors fall simultaneously within the top 10% of all the three percentile ranks (retention, source and sink), while only nine fall in the top 20% and are therefore assigned the highest community connectivity scores. It is worth noting that some sectors (5, 6 and 27) are among the top 10% with respect to one or two percentile ranks, but outside the top 20% with respect to the others, and are therefore excluded from the set of the best sectors. Vice versa, three sectors (Gallipoli, Mali Lošinj and Žirje) are among the top 20% of all percentile ranks without being in the top 10% of any. The best nine sectors are all located along the Adriatic and Ionian coasts of Apulia (Fig. 5b) and between the Kvarner Gulf and northern Dalmatia (Fig. 5c), with the top six sectors (Brindisi, Torre Canne, Bari, Torre Guaceto, Mola di Bari and Casalabate) encompassing an almost continuous region of Adriatic Apulia. Very interestingly, some of those hotspots of community connectivity are in close proximity to (or even encompass) existing protected areas, such as Torre Guaceto and Porto Cesareo MPAs in Italy, Prvić natural reserve and Kornati national park in Croatia.

## Discussion

We developed a novel methodological framework to characterize seascape connectivity at the community level. The proposed approach combines multi-species biophysical models (based on a high-resolution habitat distribution model and a realistic description of key life-history traits affecting propagule dispersal) to obtain metrics that characterize the functional role of different sites in determining patterns of community connectivity.

Within the Adriatic, complex connectivity patterns emerge at both species-specific and community levels. At the level of the single species, retention potential is quite common all over the suitable areas, but coexists with intense movement of propagules across the basin. Sectors acting as important propagule sources are in many (yet not all) cases also important sinks. Short-range connections are generally the most effective and persistent, although long-ranged species, such as *S. salpa* and *P. oceanica*, can disperse propagules over distances up to 500 km and farther (comparable with reported dispersal ranges^31^ for the genus *Posidonia*). Both *S. porcus* and *S. ocellatus* reproduce between late spring and summer, but the larval phase lasts about one month in the first species, and only ten days in the latter. On the other hand, the larval phases of *S. salpa* and *S. porcus* have approximately the same duration, but reproduction occurs in different seasons. Differences in even one single trait can critically influence intensity and persistence of propagule exchange and produce strong differences in extent and orientation of dispersal, so connectivity patterns can hardly be generalized from one species to others.

Results at the community level clearly point out that spatiotemporal variation in magnitude and direction of the connections, as well as differences in connectivity patterns among species, are crucial in structuring community connectivity: short-ranged species act as bottlenecks of community dispersal, substantially limiting the actual strength of the connection between the two sides of the Adriatic Sea. Cowen *et al*.^32^ have suggested that coastal marine populations may not be as open as previously thought, and that larval retention may be crucial to maintain population structure, with important implications on the management of marine resources. Similar conclusions have been reached by Planes *et al*.^33^, indicating that MPA networks can sustain resident populations via both local replenishment and larval dispersal from other reserves.

To date, few studies have tried to use information about dispersal patterns of marine organisms to devise criteria for marine reserves design. Shanks *et al*.^34^ reviewed dispersal distances in 32 taxa at the global scale, concluding that reserves of 4–6 km in diameter should be large enough to contain the larvae of short-distance dispersers, and reserves spaced 10–20 km apart should be close enough to capture propagules released from adjacent reserves. Using site-selection algorithms, Sala *et al*.^35^ determined that the distance between adjacent reserves in the Gulf of California should not exceed 100 km. Our results show that connections over distances comprised between 50–200 km can be very effective in this area of the Mediterranean Sea, at least for the model community considered. However, our analysis also suggests that the optimal spacing of reserves very much depends upon a variety of factors that makes it difficult to derive a rule of general validity. Seascapes are highly heterogeneous and anisotropic environments: consequently, the optimal spacing among marine reserves ultimately depends upon both the community and the habitat of interest, the specific geographical domain considered, and the relative position of candidate sites within the ocean circulation system.

Effective design of reserve networks often requires a trade-off between ensuring connectivity and providing an appropriate representation of biological diversity^36^. The Mediterranean MPA system falls short in meeting conservation targets for taxonomic, phylogenetic and functional diversity of coastal fish^37^, and the same conclusion possibly applies to other ecological compartments, including habitats. To support biodiversity protection, MPAs must concurrently be self-sustaining and linked to other protected areas so as to promote recovery from local extinctions. Although demonstrated here for a model community of up to four species only, our methodological framework is highly scalable and allows the identification of areas that act, at the same time, as best retainers, best sources and best sinks of propagules also for assemblages composed by a higher number of species. It thus provides a valuable tool to identify hotspots of metacommunity connectivity and preliminarily guide site selection, marking an important step towards a holistic approach to conservation.

Conservation priorities suggested by our study should of course be taken with a certain caution, as some factors may modify the picture emerging from the modelling results. First, although the spatiotemporal settings of the simulations represent in a credible manner the dispersing features of the species composing the model community, propagule movements from one sector to another can be affected by a range of physical and biological factors that, at present, cannot be explicitly incorporated into the model due to the lack of critical information. The reliability of our assessment may also be influenced by the resolution of the ocean circulation model. While the model used in this work provides a good description of circulation at the basin scale, it cannot reproduce water movement at the very local scale, which surely affects propagule release from source sites and their settlement into sink sites. In this sense, models capable of accurately describing water circulation near coasts, as well as small-scale fronts, eddies and gyres, would greatly enhance the realism of the simulations, especially for species, like *S. ocellatus*, whose larval dispersal is mostly confined to inshore waters. However, ocean models at such a fine spatial resolution are available only for geographical domains much smaller than ours. A trade-off is therefore needed between a realistic description of local phenomena and a good understanding of the processes influencing connectivity at the basin scale. On the biological side, our results refer to a simplified species assemblage and are therefore unable to account for the entire variety of dispersal patterns that shape realized community connectivity. In particular, species with active dispersal during any life-history stage may be able to reach suitable habitats also when physical connectivity is relatively weak. With respect to our specific case study, none of the fish species considered is exclusively linked to *P. oceanica* meadows; therefore, short-dispersing species such as *S. ocellatus* may use patches of different habitats (in spite of a lower suitability) as stepping stones allowing them to disperse across a wider range. On the other hand, the propagules of some species may remain very close to their place of birth, in spite of a relatively long larval duration, due to specific behavioural traits affecting the realized dispersal^38^,^39^.

Realized fluxes of propagules also depend upon the actual abundance and fecundity of source populations^40^; at the same time, recruitment success in destination sites can be affected by the abundance of the receiving populations through density-dependent effects^41^. Much evidence suggests that populations can be recruitment-limited at low recruit densities, whereas density-dependent post-settlement processes predominate at high densities, with recruitment variability having little influence on adult abundance. Taking these processes explicitly into account would require the development of spatially explicit metacommunity models^42^, a further modelling effort which is certainly worth but is beyond the scope of our study. Ecological interactions are another important factor influencing metacommunities, as connectivity can synchronize and amplify population fluctuations across large spatial scales with possible destabilizing effects on metacommunity dynamics^43^. Designing networks of marine reserves for ecosystem conservation requires a balance between promoting the abundance of previously exploited species and avoiding possible negative impacts of trophic cascades and competitive effects triggered by the increase of predators and competitors^44^. Despite the crucial role of intra- and interspecific interactions in the regulation of metacommunity dynamics, the explicit integration of these processes into comprehensive modelling frameworks aimed to support marine spatial planning is still in a pioneering phase, and has so far been limited to theoretical studies. Further improvements to our modelling approach would certainly include analyses borrowed from the increasingly expanding field of complex network theory science^45^,^46^,^47^,^48^,^49^.

In spite of such limitations, our results provide a new contextualization of the current knowledge about oceanographic, demographic and genetic connectivity in different regions of the Adriatic^23^,^50^,^51^,^52^ into a broader framework. The proposed methodology can be used as a starting point to characterize different aspects of connectivity, looking not only at average propagules fluxes, but taking explicitly into account their variability over time. By focusing on multiple species, we encompassed the variety of patterns that can emerge in the same physical system from differences in basic dispersing traits, allowing the assessment of connectivity at the community level, and promoting the transition from single-species to multi-species conservation targets. Our approach is, therefore, a first step toward a process-oriented framework for the design of MPA networks, in which sites can be prioritized on the basis of their functional role in shaping community connectivity.

## Methods

### Study area

**Figure 1:**
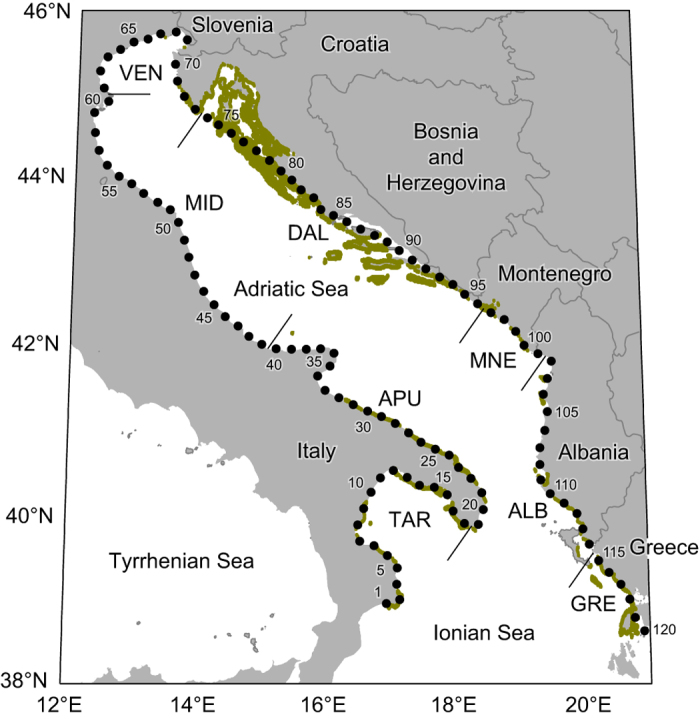
Study area. The coastline is subdivided into sectors (indicated by black dots), numbered clockwise from 1 (Capo Rizzuto, Italy) to 120 (Lefkada, Greece). Sectors are grouped into 8 regions, whose limits are indicated by thin segments (TAR: Gulf of Taranto; APU: Adriatic Apulia; MID: Middle Adriatic; VEN: Venice Lagoon; DAL: Dalmatia; MNE: Montenegro; ALB: Albania; GRE: Greece). The shading shows the suitable range for *Posidonia oceanica* (including a 2-km buffer to increase visibility) from the MediSeH project^58^ (see also Fig. 1 in Telesca *et al*.^59^). Map created with QGIS version 2.8 (www.qgis.org); country boundary lines (version 3.0.0) from Natural Earth (www.naturalearthdata.com).

**Figure 3:**
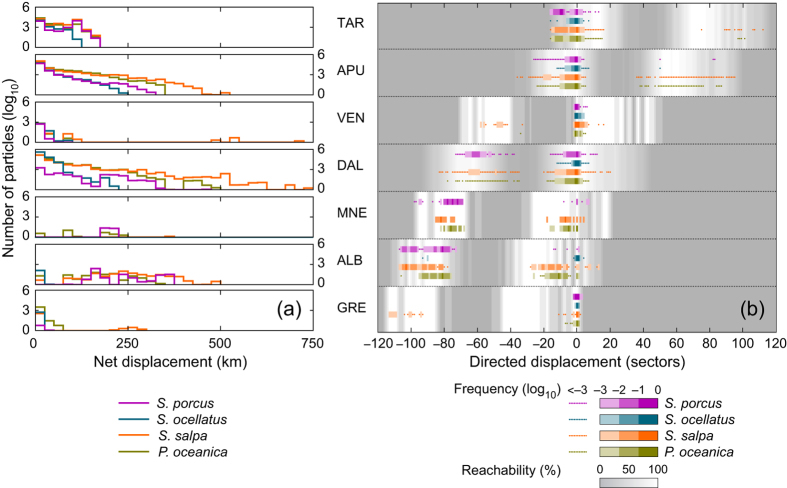
Dispersal kernels of the four species. Dispersal is evaluated for each region (see Fig. 1 for acronyms) containing suitable habitat for at least one species. (**a**) Net displacement (i.e. shortest distance within the sea from the origin to the destination) and (**b**) directed displacement of propagules (i.e. difference between the numbers identifying the sector of origin and destination, respectively; see Supplementary Fig. S1). The proportion of propagules arriving to sectors placed at different distances from the origin is expressed by colour intensity (values <10^–3^ are indicated by dots). The white to gray shaded background indicates reachability: the intensity of gray is proportional to the fraction of destination sectors at a given distance that cannot be reached, either because the habitat is unsuitable or because it is outside the model domain.

**Figure 4:**
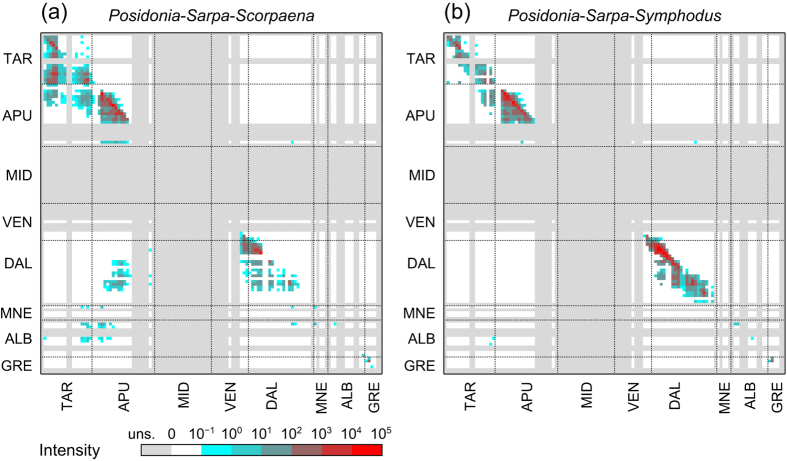
Intensity of community connectivity. (**a**) Species assemblage including *Posidonia oceanica*, *Sarpa salpa* and *Scorpaena porcus*. (**b**) Species assemblage including *P. oceanica*, *S. salpa* and *Symphodus ocellatus*. Matrix rows (columns) indicate source (sink) sectors. Uns.: unsuitable habitat.

**Figure 5:**
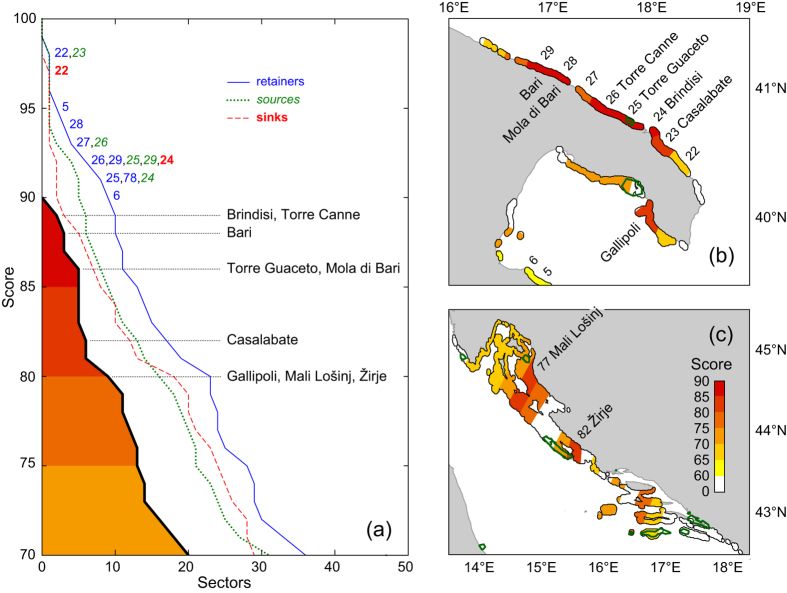
Community connectivity scores. (**a**) Cumulative distribution of percentile ranks for different functional roles (coloured lines) and community connectivity score (black), as defined in the Methods section. Panels on the right show the sectors with the highest community connectivity score along Apulian (**b**) and Croatian (**c**) coasts (see Supplementary Fig. S21 for the map of the entire basin). The level of the score is indicated by the shading (from yellow to red), while green contours show existing protected areas. Sectors falling within the 10th percentile rank of any functional role are indicated by their corresponding numbers (see Fig. 1), while those with the highest community connectivity score (between 80 and 90) are indicated by their toponyms. Map created with QGIS version 2.8 (www.qgis.org).

The Adriatic Sea (Fig. 1) is the northernmost arm of the Mediterranean Sea, extending from the Gulf of Venice to the Strait of Otranto, where it connects with the Ionian Sea. Water circulation (Fig. S2) is characterized by a large cyclonic meander, with prevailing currents flowing northwards along the eastern coast and southwards along the western one^53^. Smaller-scale circulation is characterized by three minor gyres, subject to seasonal and short-term variability^50^. The oceanographic subdivision of the Adriatic into three sub-regions, connected by a major circulation system and a series of ephemeral connections, influences seascape connectivity within the basin, as suggested by recent comparative studies integrating population genetics, analysis of fisheries data, otolith microchemistry and biophysical modelling^52^,^54^.

### Model community

We considered simplified model communities composed by different assemblages of three/four species including: one primary producer (*P. oceanica*), one primary consumer (*S. salpa*) and one/two secondary consumers (*S. ocellatus* and/or *S. porcus*). Although extremely simplified, the studied assemblages are representative of a typical Mediterranean ecosystem, since the three species are closely associated with *Posidonia* meadows, where they are remarkably more abundant than in other habitats. The most important dispersing stages are fruits for *P. oceanica*^55^ and larvae for the fishes^56^. While all three fish species inhabit *P. oceanica* meadows, their depth ranges overlap only partially and they are characterized by different dispersal strategies. Biological information about key dispersing was collected from the literature (see Supplementary Information).

### Lagrangian simulations

We used daily average current velocity fields produced by the Adriatic Forecasting System (http://gnoo.bo.ingv.it/afs) using the Adriatic Sea Regional Model^57^. Suitable conditions for *P. oceanica* were derived from a suitability model developed in the framework of the MediSeH project^58^,^59^, a joint Mediterranean-wide effort that has produced the most up-to-date information on the distribution of seagrass meadows. The suitable habitats for the other species were obtained by intersecting the distribution range of *P. oceanica* with the depth range in which each species is known to occur. In all simulation years (from 2003 to 2013), Lagrangian particles representing dispersing propagules were released during the species-specific reproduction season from the appropriate suitable habitat and they were tracked for the duration of the corresponding dispersing stage. To achieve statistical significance in the calculation of connectivity between different locations, the number of released propagules was in the order of millions (see Supplementary Information for further details). A propagule was considered successful if it arrived into a suitable location at the end of the dispersing phase.

### Dispersal kernels

The coastline of the study area was preliminarily approximated with a polygonal chain of 25-km long segments (a trade-off between spatial resolution and precision of connectivity estimates). The segments were used as a basis to subdivide the area into 120 sectors (Fig. 1). As the extent of suitable habitat differs from one sector to another and from one species to another, the number of propagules originating from each sector varies accordingly, and no propagule were obviously released from unsuitable sectors. Each sector was then associated to one of 8 regions, corresponding to major geographical and/or administrative units. For each species and each region, we derived two dispersal kernels: one measuring the net displacement (*sensu* Turchin^30^, i.e. the shortest distance within the sea) of propagules from the origin to the destination; the other measuring the directed displacement (in sectors) along the coastline, i.e. the difference between the numbers identifying the sector of origin and destination, respectively (Supplementary Fig. S1).

### Species-specific connectivity metrics

For each species and each year *t* of our Lagrangian modelling, we calculated a flow matrix Φ_*t*_ whose element *ϕ*_*ij*,*t*_ represents the number of propagules starting from source sector *i* in year *t* and successfully arriving to sink sector *j* within the duration of the relevant dispersing phase (*i*, *j* = 1, 2, … *N*; *t* = 1, 2, … *T*, where *N* = 120 is the number of sectors and *T* = 11 the number of years covered by the simulations). Connectivity between sectors was characterized by three sets of metrics, providing substantial and complementary information to quantify connectivity between sectors: intensity, effectiveness and persistence. We produced a connectivity matrix for each of the three metrics. Intensity (*I*) was obtained by averaging the flow matrix over time, i.e. 
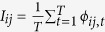
. Effectiveness (*E*) was defined as the ratio between intensity *I* and the capacity to originate (or receive) propagules. As releases were uniformly distributed across the suitable area, effectiveness was calculated as intensity per unit of suitable area. Finally, persistence (*P*) was defined as the stabilization coefficient (*sensu* Liu & Zheng^60^) of the flow matrix over years, i.e. 
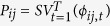
. The stabilization coefficient 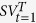 is the reciprocal of the coefficient of variation of *ϕ* over time (calculated over the period *t* = 1, 2, … *T*).

To characterize the capacity of a sector to serve as a suitable retainer, source or sink for the propagules of a given species, we defined three indicators of intensity, effectiveness and persistence for each sector *k*. For intensity,


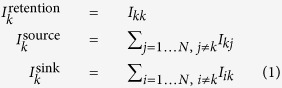


where 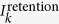 represents the number of propagules released in sector *k* and retained within it, 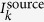 is the number of propagules starting from sector *k* and successfully arriving to any other sector, and 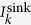 is the number of propagules successfully reaching sector *k* from any other sector. For effectiveness,


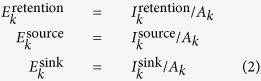


For persistence,


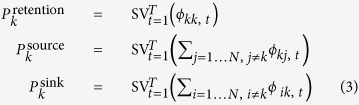


Each sector was therefore characterized by 3 × 3 species-specific indicators quantifying its functional role (retainer, source or sink) along three major axes of connectivity (intensity, effectiveness and persistence). It is important to notice that our measures of connectivity are estimates of potential connectivity, as they do not account for the complex suite of pre-settlement events able to affect the realized connectivity. These include, in particular, the actual number of propagules generated from each patch and their probability to survive the dispersal phase and successfully settle into the destination site, as well as the effect of anthropogenic pressure on realized habitat suitability.

### From species-specific indicators to community connectivity indices

Connectivity matrices and indicators were derived for each species. As our final aim was to obtain measures of connectivity at the community rather than the population level, we aggregated species-specific indicators into community connectivity indices. To account for the fact that poor connectivity in one species cannot be completely compensated by good levels of connectivity in other species, we calculated each community index as the geometric mean of the corresponding species-specific indicators. The geometric mean is a measure of central tendency that places more weight (compared with the arithmetic mean) on the lowest values, thus providing a conservative measure of community connectivity.

### Percentile ranks and community connectivity score

To assign an overall score of community connectivity to each sector, we first elaborated the above defined community connectivity indices by independently considering the three functional roles a sector can play: retainer, source or sink. Looking at retention, for instance, we ranked all sectors in decreasing order of a) retention intensity, b) retention effectiveness, and c) retention persistence, thus obtaining three different rankings with respect to retention. Three percentile ranks were then assigned to each sector, each defined as the percentage of sectors in distributions a), b) and c), respectively, that have a rank equal or lower than that of the considered sector. We named *retention percentile rank* the minimum of the three percentile ranks related to retention. An analogous reasoning was followed to define a *source percentile rank* and a *sink percentile rank*. Eventually, each sector was assigned a *community connectivity score*, defined as the minimum among the three (retention, source and sink) percentile ranks.

## Additional Information

**How to cite this article**: Melià, P. *et al*. Looking for hotspots of marine metacommunity connectivity: a methodological framework. *Sci. Rep*. **6**, 23705; doi: 10.1038/srep23705 (2016).

## Supplementary Material

Supplementary Information

## Figures and Tables

**Figure 2:**
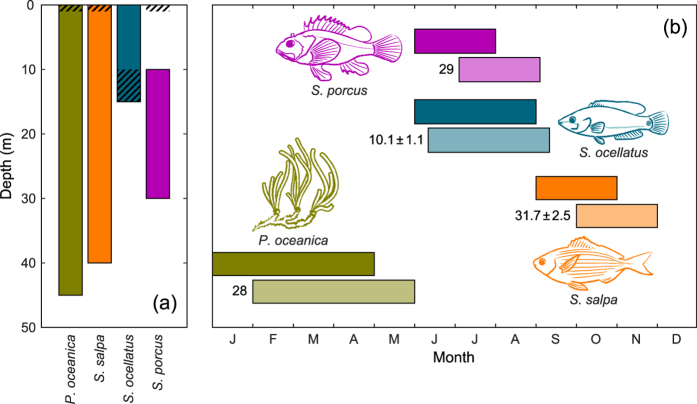
Spatiotemporal features affecting dispersal of the model community. (**a**) Suitable depth range for recruitment (bars) and dispersal depth of propagules (hatched areas) of each species (colour-coded). (**b**) Timing of propagule dispersal: period of propagule release (upper bar) and recruitment (lower bar). The recruitment period is obtained by adding the average duration of the dispersing stage (indicated by numbers to the left of the lower bar, ±SD when available) to the period of propagule release.
