# Supplementary material for: Looking for hotspots of marine metacommunity connectivity: a methodological framework
Source: Sci Rep. 2016 Mar 31;6:23705. doi: 10.1038/srep23705 (PMC4814777; doi:10.1038/srep23705)
Supplement: Supplementary Information [file srep23705-s1.pdf]

Looking for hotspots of marine metacommunity connectivity: a methodological framework

P. Melià, M. Schiavina, M. Rossetto, M. Gatto, S. Fraschetti and R. Casagrandi

Supplementary Information

Supplementary methods

Dispersing features of the species

1. The marine angiosperm *Posidonia oceanica* is the dominant seagrass in the Mediterranean Sea and forms large and stable meadows at depths between 0–45 m[1] providing suitable habitat for many marine organisms[2]. *P. oceanica* produces positively buoyant fruits, which are occasionally released between January and April[3,4] and float for about 28 days before releasing their seed[5].
2. The salema porgy *Sarpa salpa* inhabits seagrass meadows at depths between 0–40 m[6,7] and is a primary grazer, accounting for 40–70% of the fish biomass and up to 75% of total herbivorous consumption in *P. oceanica* meadows[8–10]. Although *S. salpa* can reproduce both in spring and autumn[11], in many regions spawning takes place mainly or exclusively in autumn. In the Adriatic Sea, it is typically concentrated between September and October[12]. Pelagic eggs hatch 2 days after release; larvae are also pelagic and drift for 27–38 days[11].
3. The ocellated wrasse *Symphodus ocellatus* is the most abundant representative of the family Labridae in *P. oceanica* meadows of the Adriatic Sea[13]; it is an invertebrate feeder[14] found at depths ranging from 1 to 15 m[15,16]. In the Adriatic Sea, *S. ocellatus* lays benthic eggs between July and August[12]; larvae last 8–13 days in the water column before settling[11].
4. The black scorpion fish *Scorpaena porcus* is a small piscivorous fish[14] commonly found in inshore waters among rocks and seagrass beds between 10–30 m depth[17]. Pelagic eggs are released between June and July[12], float at the water surface and hatch after about 2 days[18]; larvae last 29 days in the water column[11].

Lagrangian simulations

The physical engine used to run the biophysical models is the Adriatic Sea Regional Model (Oddo et al., 2006), characterized by a horizontal resolution of 1/45° (about 2.2 km) and 31 vertical sigma layers. The spatial domain of the model encompasses the whole Adriatic basin and the northernmost part of the Ionian Sea down to the 39°N parallel; the bathymetry has a horizontal resolution of 1/60°, with the coastline set in correspondence with the 10-m isobath.

Lagrangian simulations were run using current velocity fields for the period 2003–2013. Lagrangian particles were released from the suitable habitat of each species and tracked for the duration of the corresponding dispersing stage. Releases were performed at a uniform density, assuming that reproductive life stages (flowering plants and fish spawners) are evenly distributed over the whole suitable habitat. The density of release was set to 2000 particles per km^2 of suitable area (allowing good coverage of the habitat while ensuring a reasonable computational load). As the extent of the suitable habitat was different for each species, the actual number of particles released varied accordingly, ranging approximately between 1 million per year (*S. porcus*) and 5 million per year (*P. oceanica*). Each particle was assigned a fixed depth layer between 0–1 m for *P. oceanica* fruits and pelagic fish larvae, and between 10–15 m for *S. ocellatus* larvae. The geographic coordinates and the depth from which each particle was released, along with the day of release, were randomly drawn from (multivariate) uniform distributions, while the duration of the dispersing phase was extracted from a Gaussian distribution with mean and standard deviation matching the specific reproductive period documented in the literature (see Fig. 2 in the main text).

Trajectories were stepped forward via a 4th-order Runge-Kutta integration scheme with a 6-minute time step and followed until the end of the dispersing phase. Local current velocity was derived via a linear convex combination in space and a linear interpolation in time of the current velocity field provided by the oceanographic model.

Supplementary references

1. Pasqualini, V., Pergent-Martini, C., Clabaut, P. & Pergent, G. Mapping of *Posidonia oceanica* using aerial photographs and side scan sonar: application off the island of Corsica (France). *Estuar. Coast. Shelf Sci.* **47,** 359–367 (1998).

2. Giakoumi, S. *et al.* Towards a framework for assessment and management of cumulative human impacts on marine food webs. *Conserv. Biol.* **29,** 1228–1234 (2015).

3. Buia, M. C. & Mazzella, L. Reproductive phenology of the Mediterranean seagrasses *Posidonia oceanica* (L.) Delile, *Cymodocea nodosa* (Ucria) Aschers., and *Zostera noltii* Hornem. *Aquat. Bot.* **40,** 343–362 (1991).

4. Balestri, E. & Cinelli, F. Sexual reproductive success in *Posidonia oceanica*. *Aquat. Bot.* **75,** 21–32 (2003).

5. Serra, I. A. *et al.* Genetic structure in the Mediterranean seagrass *Posidonia oceanica*: disentangling past vicariance events from contemporary patterns of gene flow. *Mol. Ecol.* **19,** 557–568 (2010).

6. Verlaque, M. Relations entre Sarpa salpa (Linnaeus, 1758) (Téléostéen, Sparide), les autres poissons brouteurs et le phytobentos algal méditerranéen. *Oceanol. Acta* **13,** 373–388 (1990).

7. Méndez-Villamil, M., Pajuelo, J. G., Lorenzo, J. M., Coca, J. & Ramos, A. G. Age and growth of the salema, *Sarpa salpa* (Osteichtyes, Sparidae), off the Canary Islands (East-Central Atlantic). *Arch. Fish. Mar. Res.* **49,** 139–148 (2001).

8. Cebrián, J. *et al.* Herbivory on *Posidonia oceanica*: magnitude and variability in the Spanish Mediterranean. *Mar. Ecol. Prog. Ser.* **130,** 147–155 (1996).

9. Francour, P. Fish Assemblages of *Posidonia oceanica* beds at Port-Cros (France, NW Mediterranean): assessment of composition and long-term fluctuations by visual census. *Mar. Ecol.* **18,** 157–173 (1997).

10. Steele, L., Darnell, K., Cebrián, J. & Sanchez-Lizaso, J. *Sarpa salpa* herbivory on shallow reaches of *Posidonia oceanica* beds. *Anim. Biodivers. Conserv.* **37.1,** 49–57 (2014).

11. Macpherson, E. & Raventos, N. Relationship between pelagic larval duration and geographic distribution of Mediterranean littoral fishes. *Mar. Ecol. Prog. Ser.* **327,** 257–265 (2006).

12. Tsikliras, A. C., Antonopoulou, E. & Stergiou, K. I. Spawning period of Mediterranean marine fishes. *Rev. Fish Biol. Fish.* **20,** 499–538 (2010).

13. Guidetti, P. Differences among fish assemblages associated with nearshore *Posidonia oceanica* seagrass beds, rocky–algal reefs and unvegetated sand habitats in the Adriatic Sea. *Estuar. Coast. Shelf Sci.* **50,** 515–529 (2000).

14. Guidetti, P. & Sala, E. Community-wide effects of marine reserves in the Mediterranean Sea. *Mar. Ecol. Prog. Ser.* **335,** 43–56 (2007).

15. Harmelin-Vivien, M. L. & Francour, P. Trawling or visual censuses? Methodological bias in the assessment of fish populations in seagrass beds. *Mar. Ecol.* **13,** 41–51 (1992).

16. Macpherson, E., Gordoa, A. & García-Rubies, A. Biomass size spectra in littoral fishes in protected and unprotected areas in the NW Mediterranean. *Estuar. Coast. Shelf Sci.* **55,** 777–788 (2002).

17. Harmelin-Vivien, M. L., Kaim-Malka, R. A., Ledoyer, M. & Jacob-Abraham, S. S. Food partitioning among scorpaenid fishes in Mediterranean seagrass beds. *J. Fish Biol.* **34,** 715–734 (1989).

18. Jug-Dujaković, J., Dulčić, J. & Kraljević, M. Preliminary data on embryological and larval development of black scorpionfish Scorpaena porcus (Linnaeus, 1758). *Notes Inst. Oceanogr. Fish. Split* **78,** 1–7 (1995).

Supplementary figures

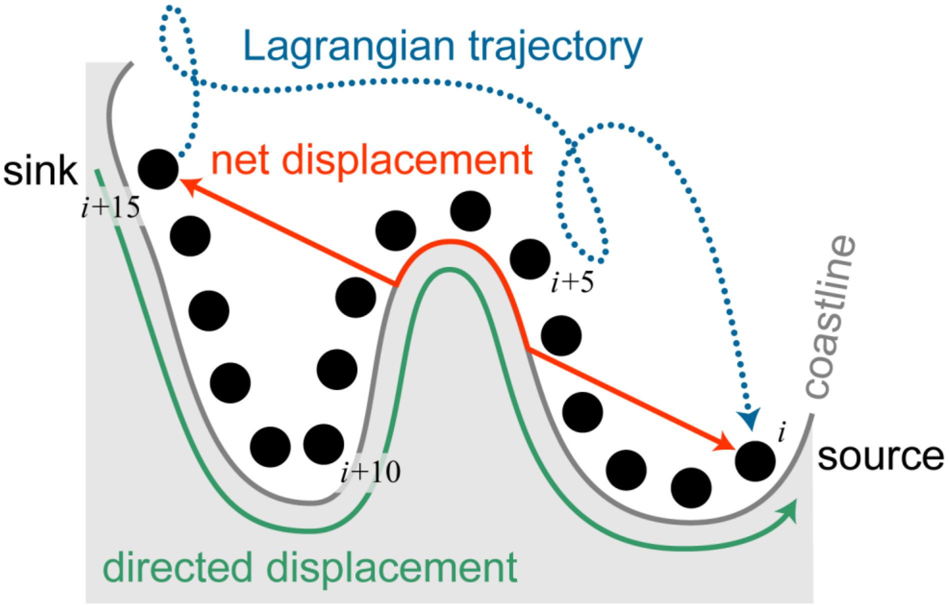

Fig. S1 | Measures of propagule displacement used to derive dispersal kernels. Circles represent the sectors into which the coastline (grey line) has been subdivided. The blue, dotted line shows the hypothetical trajectory of a particle (simulated via Lagrangian simulation) from a source sector to a sink sector (black dots). The red line indicates the *net displacement* of the particle, defined as the length (in km) of the shortest path from the source to the sink that remains within the sea (taking into account physical barriers such as headlands or islands). The green line indicates what we call the *directed displacement* of the particle, measured as the difference between the number identifying the sector of destination and that of the sector of origin. Because of the way we labelled the sectors within the study area (clockwise from Capo Rizzuto, Italy, to Lefkada, Greece), directed displacement is positive for particles moving clockwise within the Adriatic and negative for particles moving counter-clockwise. In the example, the directed displacement of the particle equals –15 sectors.

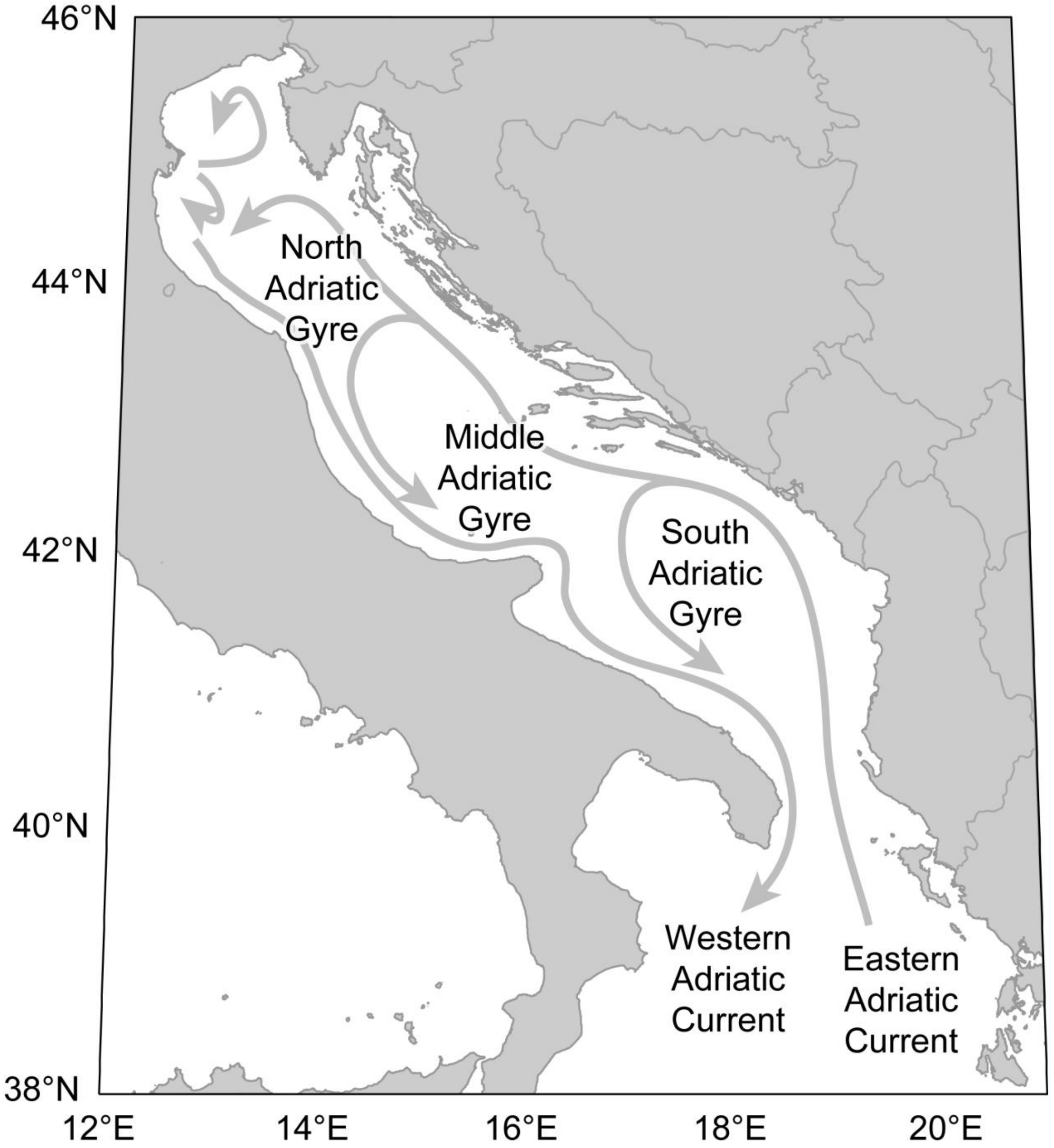

Fig. S2 | Sketch of the general surface circulation of the Adriatic Sea. Map created with QGIS version 2.8 (www.qgis.org).

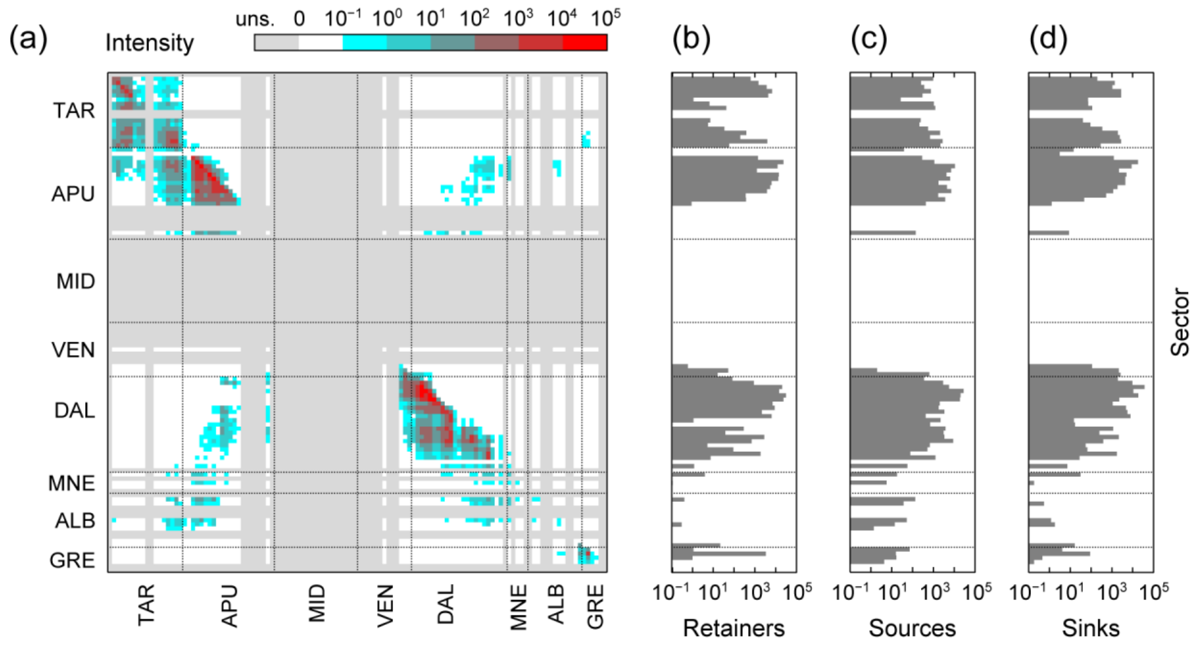

Fig. S3 | Intensity matrix for *Posidonia oceanica* (a) and histograms of retention (b), source (c) and sink intensity (d).

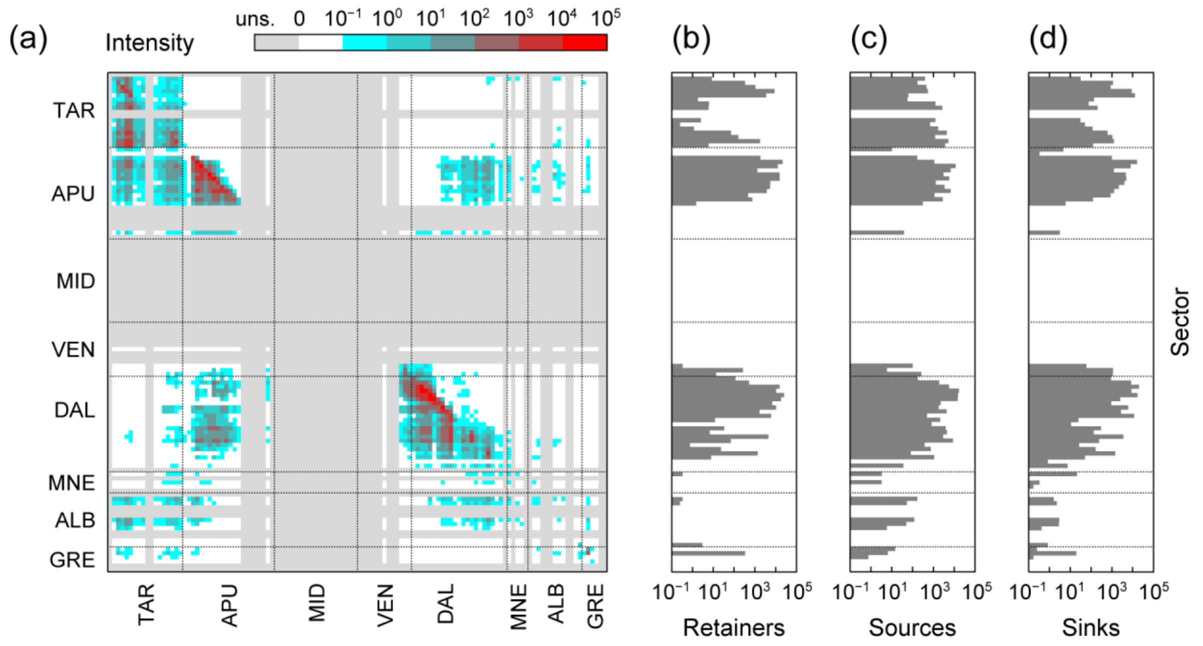

Fig. S4 | Intensity matrix for *Sarpa salpa* (a) and histograms of retention (b), source (c) and sink intensity (d).

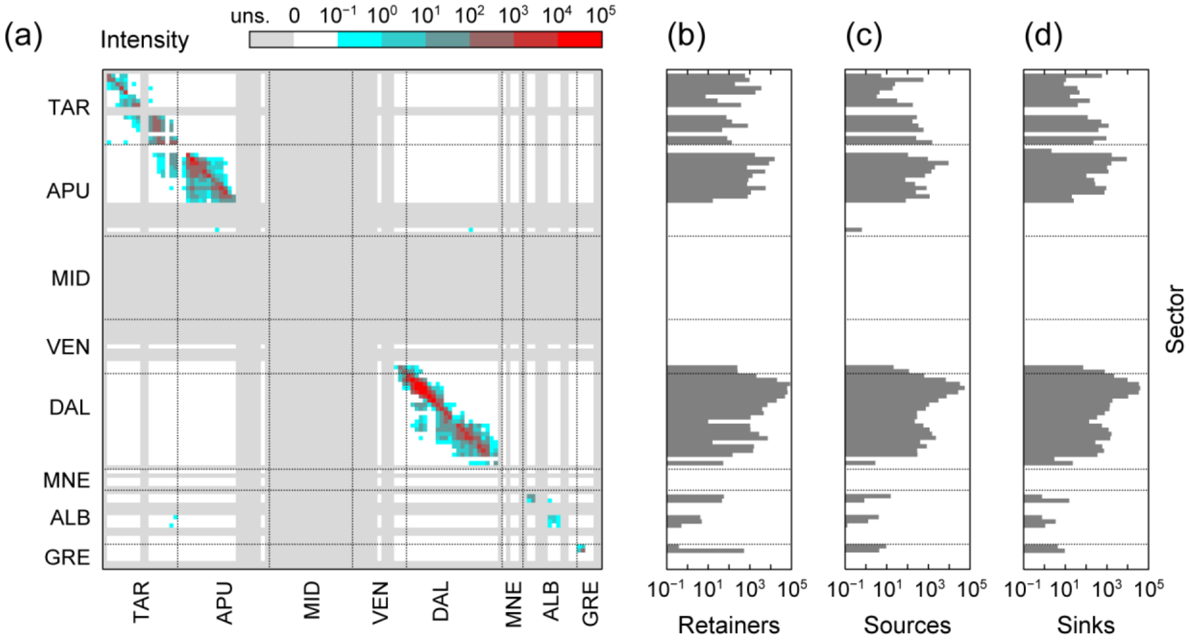

Fig. S5 | (a) Intensity matrix for *Symphodus ocellatus* (a) and histograms of retention (b), source (c) and sink intensity (d).

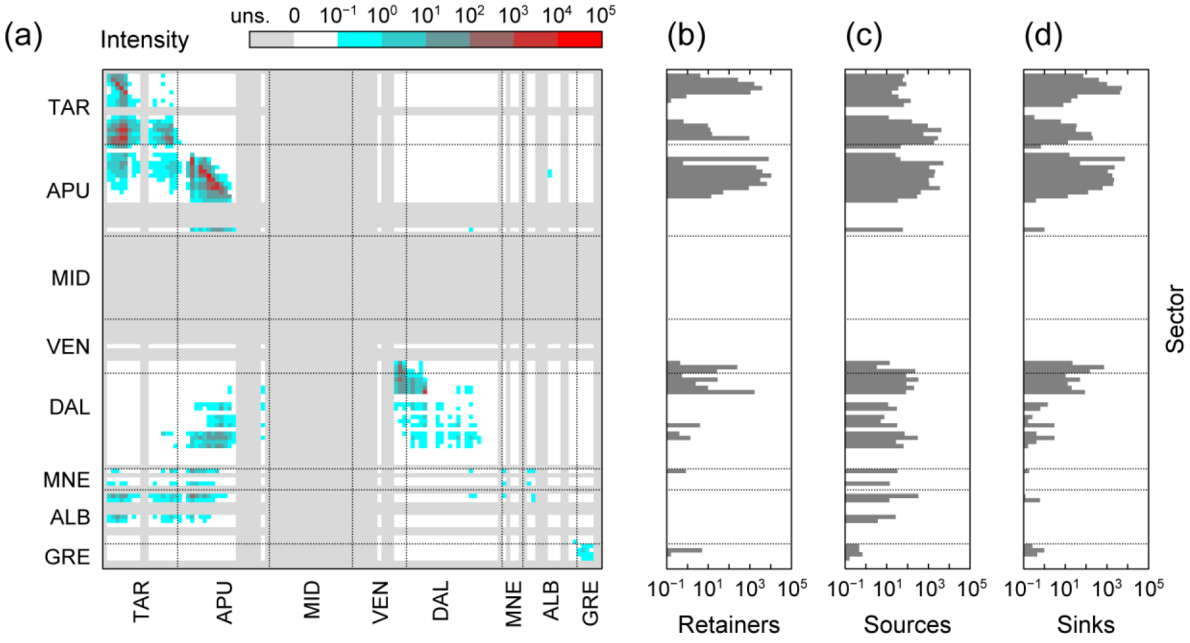

Fig. S6 | (a) Intensity matrix for *Scorpaena porcus* (a) and histograms of retention (b), source (c) and sink intensity (d).

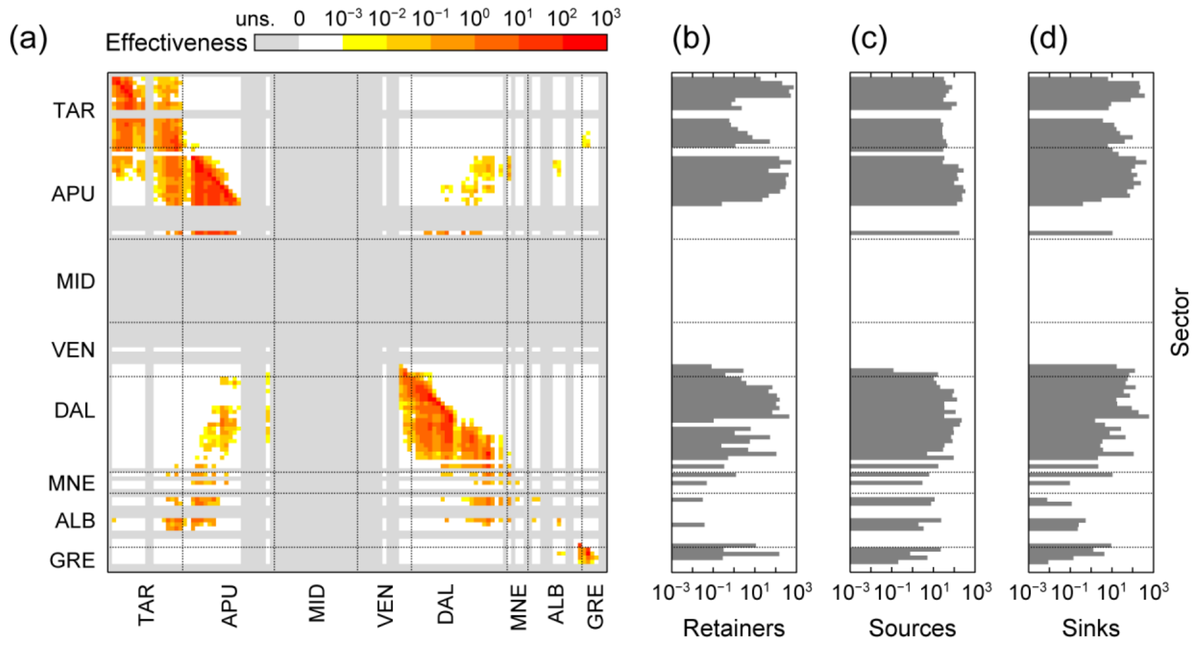

Fig. S7 | Effectiveness matrix for *Posidonia oceanica* (a) and histograms of retention (b), source (c) and sink effectiveness (d).

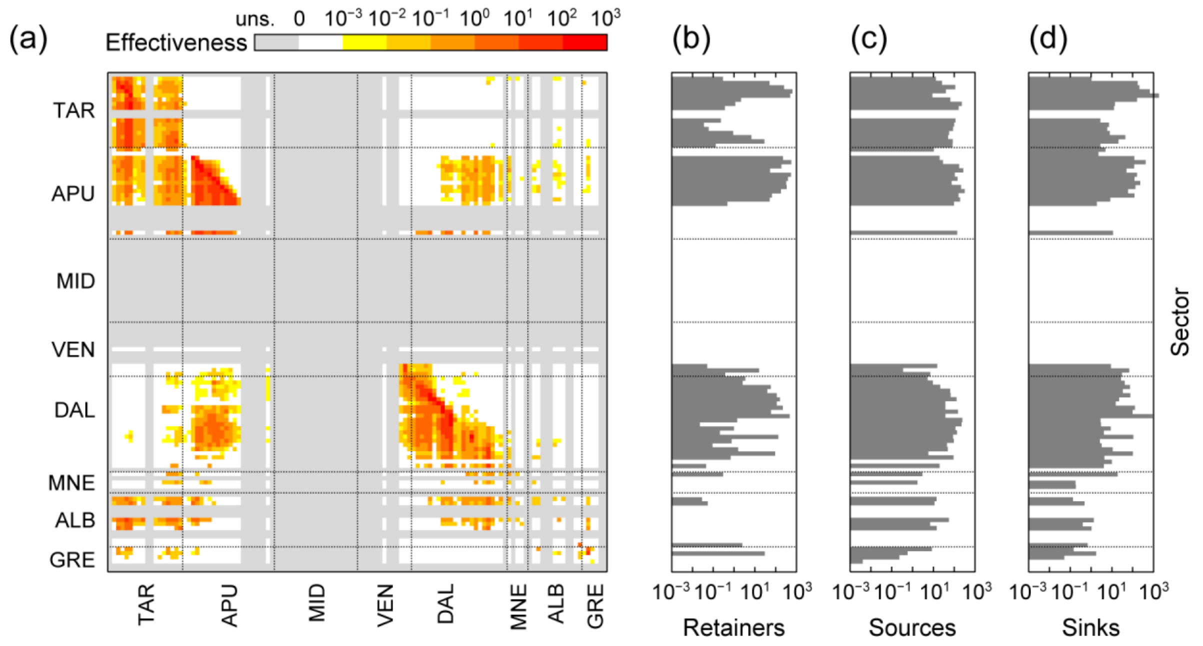

Fig. S8 | Effectiveness matrix for *Sarpa salpa* (a) and histograms of retention (b), source (c) and sink effectiveness (d).

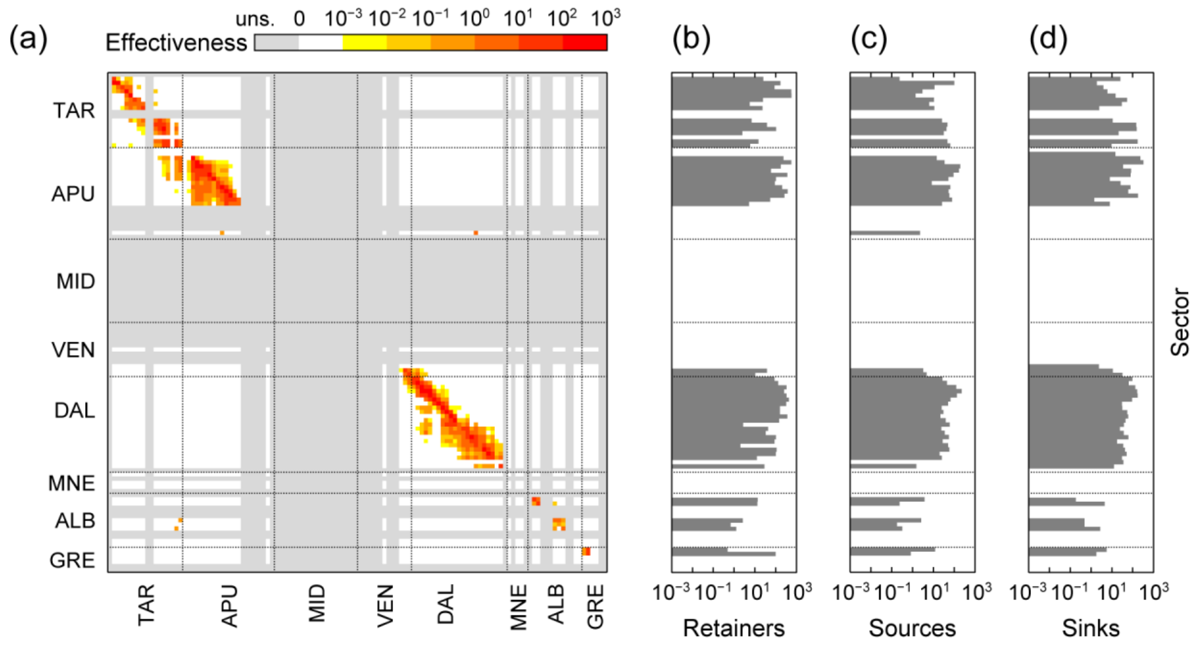

Fig. S9 | Effectiveness matrix for *Symphodus ocellatus* (a) and histograms of retention (b), source (c) and sink effectiveness (d).

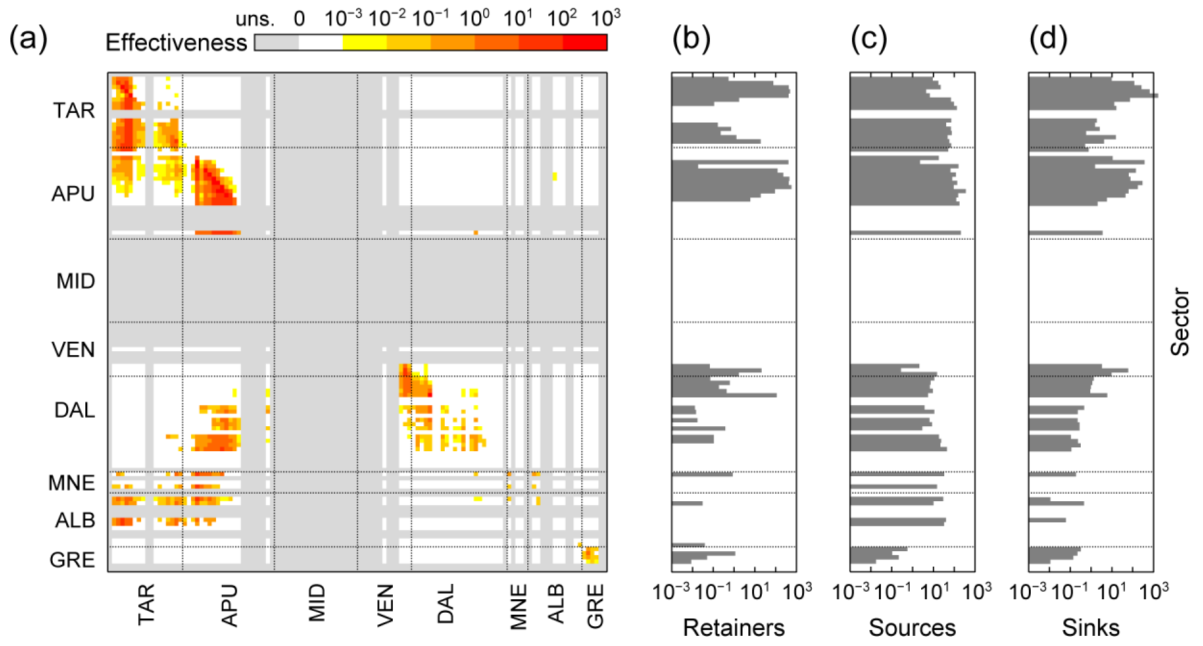

Fig. S10 | Effectiveness matrix for *Scorpaena porcus* (a) and histograms of retention (b), source (c) and sink effectiveness (d).

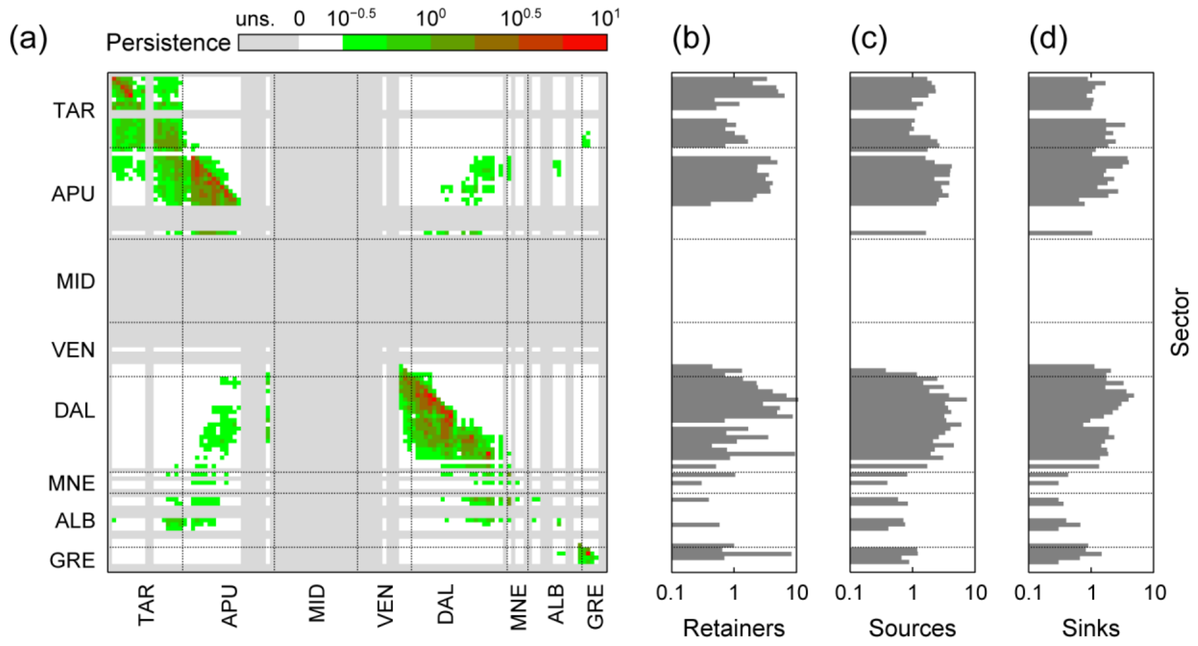

Fig. S11 | Persistence matrix for *Posidonia oceanica* (a) and histograms of retention (b), source (c) and sink persistence (d).

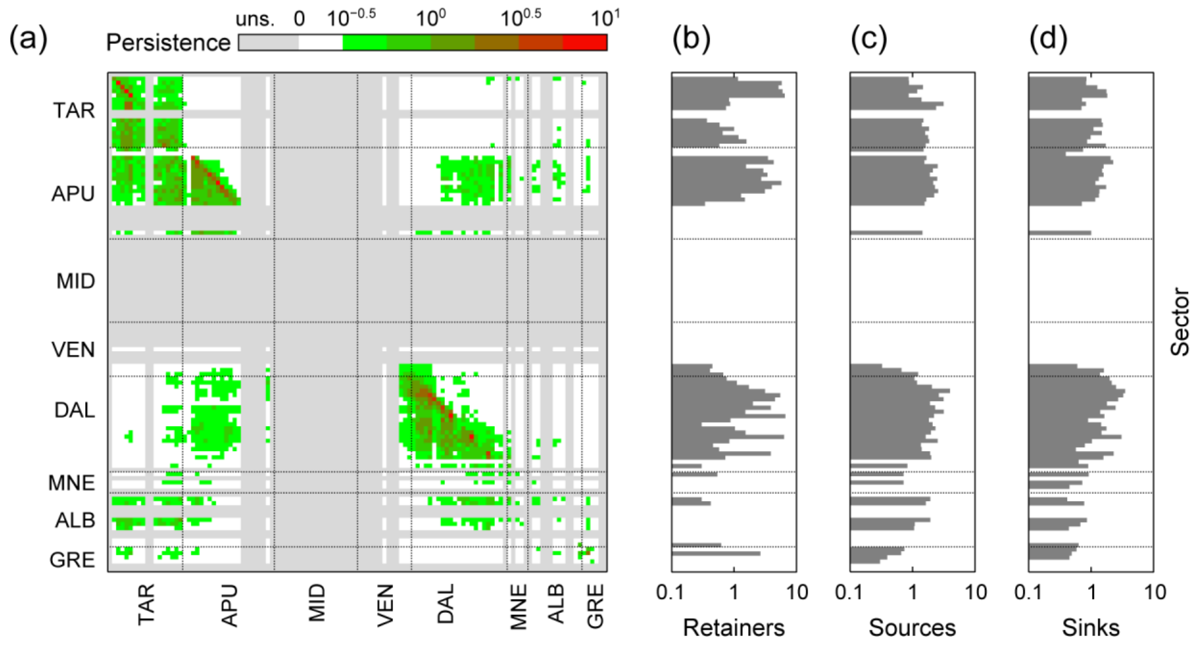

Fig. S12 | Persistence matrix for *Sarpa salpa* (a) and histograms of retention (b), source (c) and sink persistence (d).

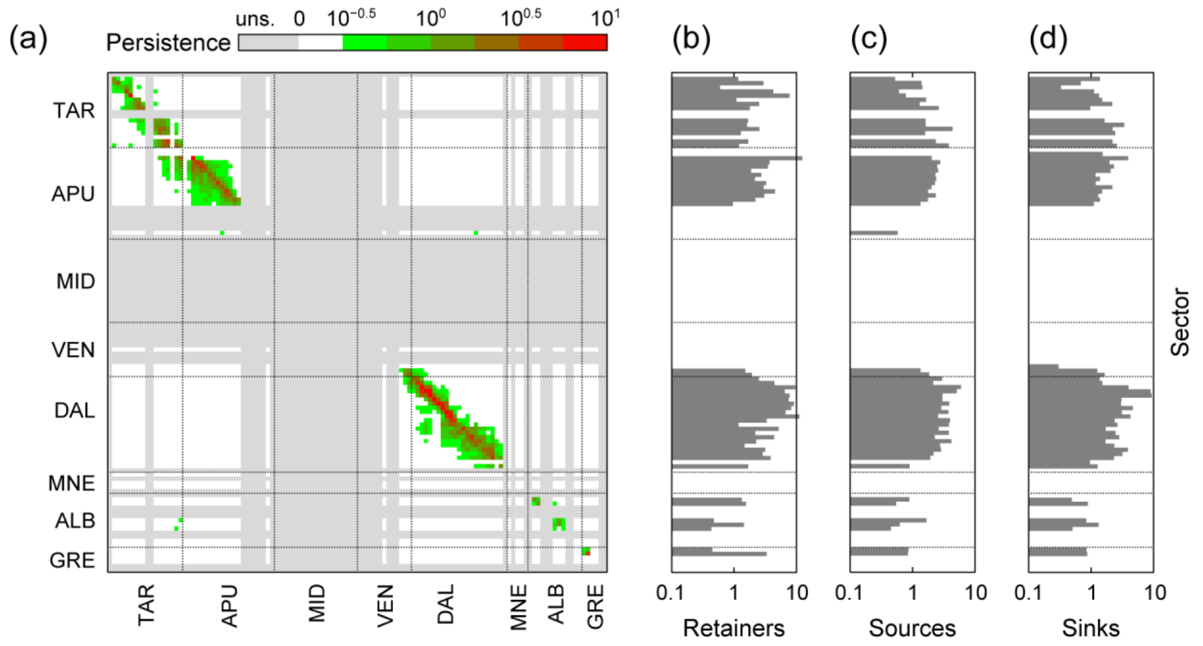

Fig. S13 | Persistence matrix for *Symphodus ocellatus* (a) and histograms of retention (b), source (c) and sink persistence (d).

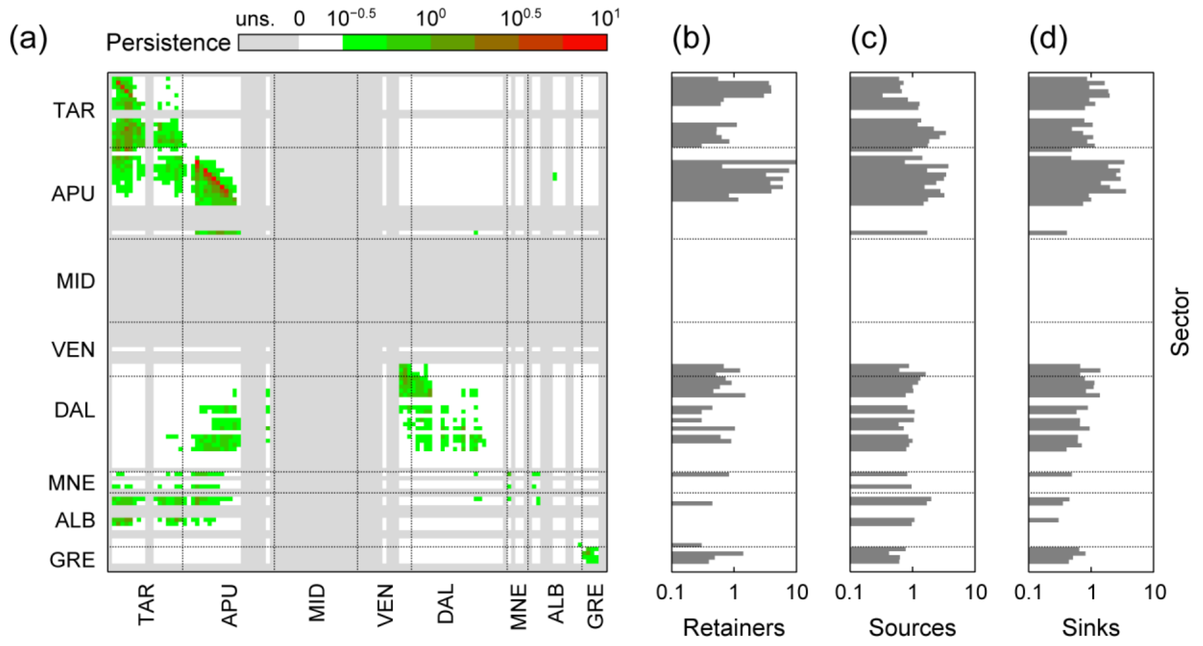

Fig. S14 | Persistence matrix for *Scorpaena porcus* (a) and histograms of retention (b), source (c) and sink persistence (d).

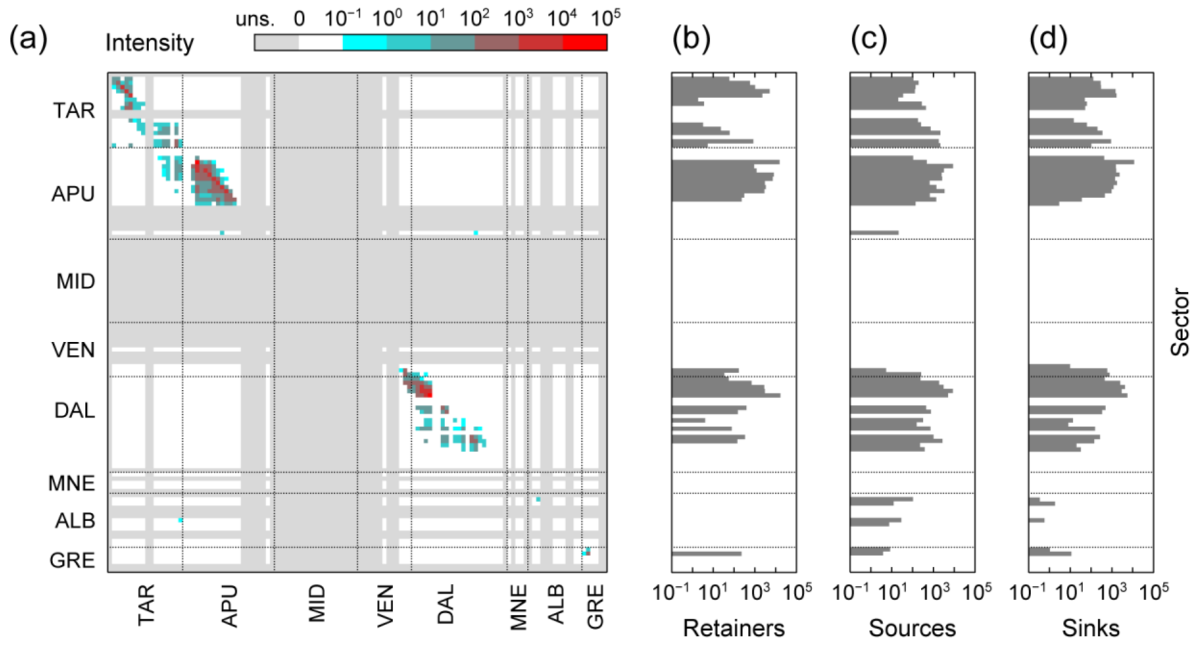

Fig. S15 | Intensity matrix for the four-species assemblage (a) and histograms of retention (b), source (c) and sink intensity (d).

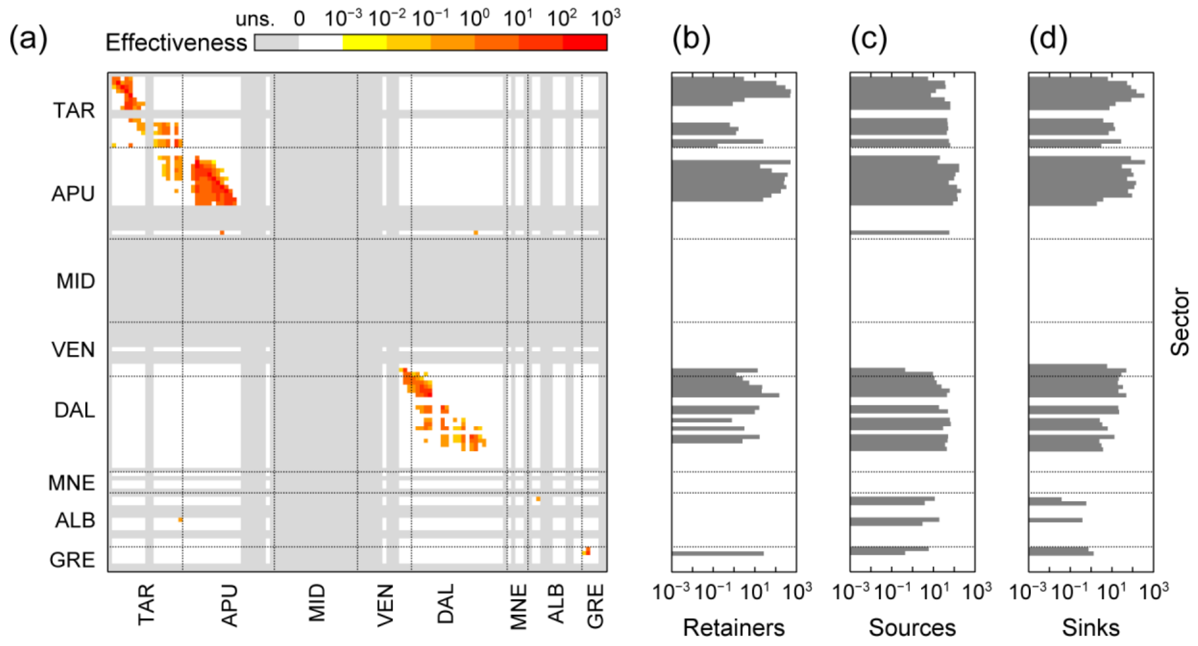

Fig. S16 | Effectiveness matrix for the four-species assemblage (a) and histograms of retention (b), source (c) and sink effectiveness (d).

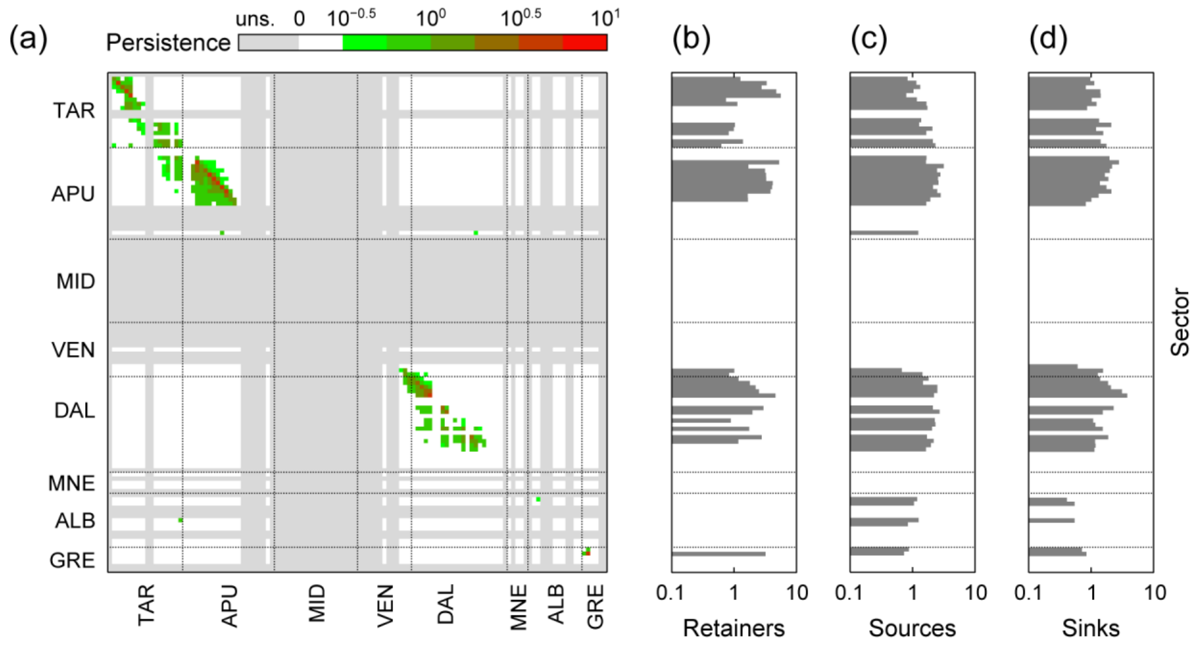

Fig. S17 | Persistence matrix for the four-species assemblage (a) and histograms of retention (b), source (c) and sink persistence (d).

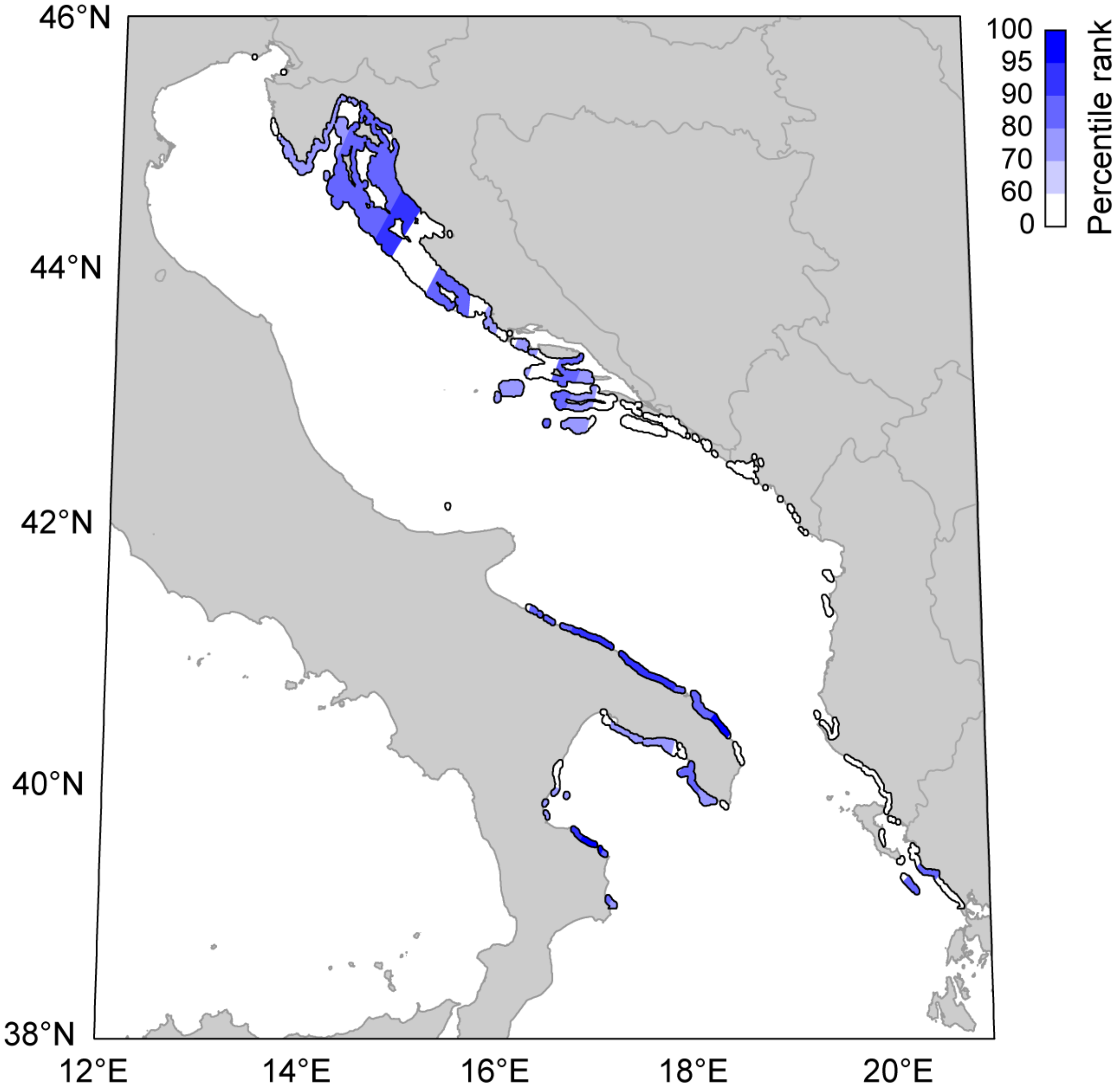

Fig. S18 | Retention percentile rank of the sectors (for the four-species assemblage). Map created with QGIS version 2.8 (www.qgis.org).

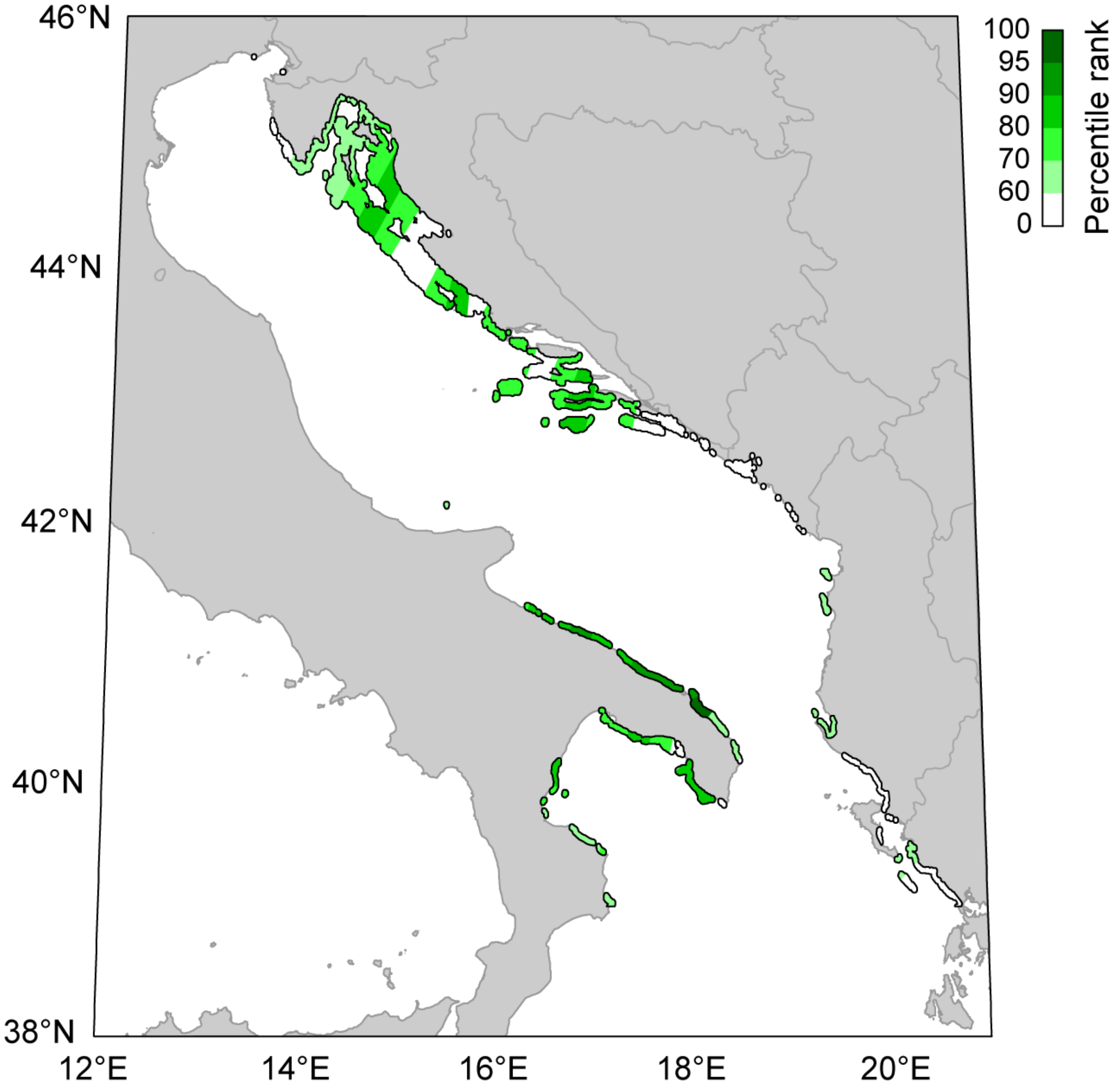

Fig. S19 | Source percentile rank of the sectors (for the four-species assemblage). Map created with QGIS version 2.8 (www.qgis.org).

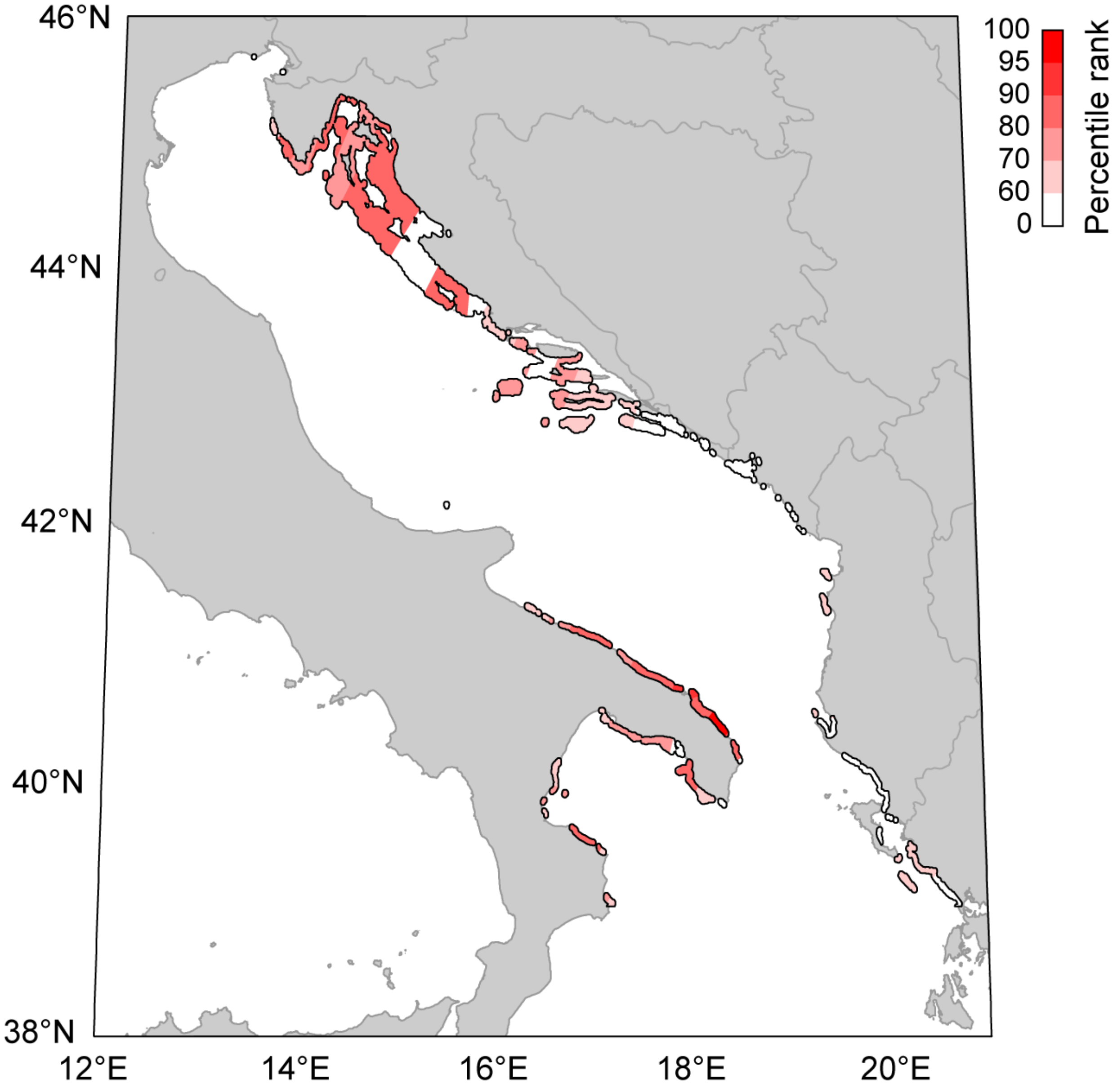

Fig. S20 | Sink percentile rank of the sectors (for the four-species assemblage). Map created with QGIS version 2.8 (www.qgis.org).

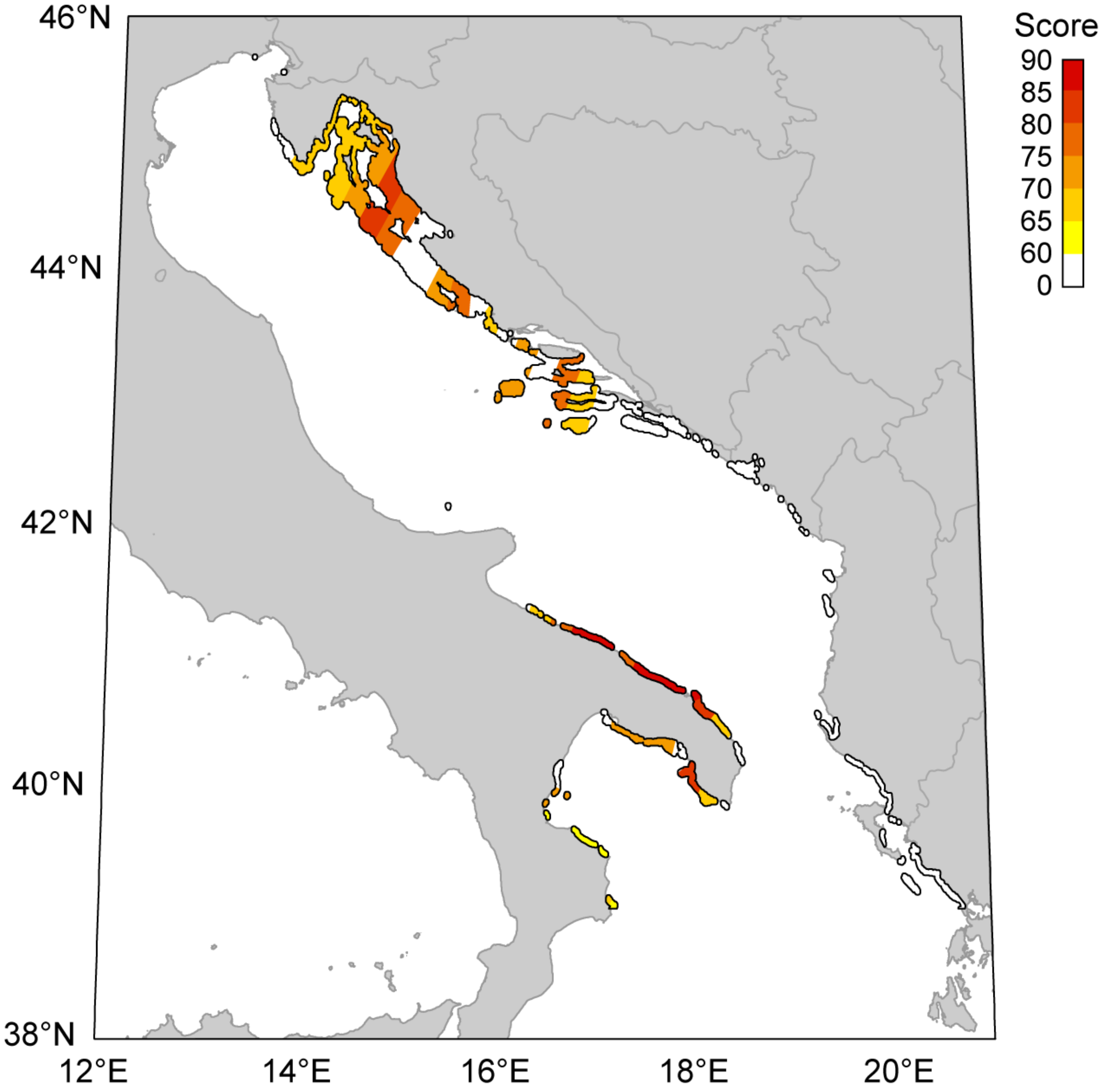

Fig. S21 | Community connectivity score of the sectors (for the four-species assemblage). Map created with QGIS version 2.8 (www.qgis.org).
